# A global experiment on motivating social distancing during the COVID-19 pandemic

**DOI:** 10.1073/pnas.2111091119

**Published:** 2022-05-27

**Authors:** Psychological Science Accelerator Self-Determination Theory Collaboration

**Keywords:** behavior change, motivation, health communication, COVID-19, self-determination theory

## Abstract

Finding communication strategies that effectively motivate social distancing continues to be a global public health priority during the COVID-19 pandemic. This cross-country, preregistered experiment (*n* = 25,718 from 89 countries) tested hypotheses concerning generalizable positive and negative outcomes of social distancing messages that promoted personal agency and reflective choices (i.e., an autonomy-supportive message) or were restrictive and shaming (i.e., a controlling message) compared with no message at all. Results partially supported experimental hypotheses in that the controlling message increased controlled motivation (a poorly internalized form of motivation relying on shame, guilt, and fear of social consequences) relative to no message. On the other hand, the autonomy-supportive message lowered feelings of defiance compared with the controlling message, but the controlling message did not differ from receiving no message at all. Unexpectedly, messages did not influence autonomous motivation (a highly internalized form of motivation relying on one’s core values) or behavioral intentions. Results supported hypothesized associations between people’s existing autonomous and controlled motivations and self-reported behavioral intentions to engage in social distancing. Controlled motivation was associated with more defiance and less long-term behavioral intention to engage in social distancing, whereas autonomous motivation was associated with less defiance and more short- and long-term intentions to social distance. Overall, this work highlights the potential harm of using shaming and pressuring language in public health communication, with implications for the current and future global health challenges.

## Significance

Communicating in ways that motivate engagement in social distancing remains a critical global public health priority during the COVID-19 pandemic. This study tested motivational qualities of messages about social distancing (those that promoted choice and agency vs. those that were forceful and shaming) in 25,718 people in 89 countries. The autonomy-supportive message decreased feelings of defying social distancing recommendations relative to the controlling message, and the controlling message increased controlled motivation, a less effective form of motivation, relative to no message. Message type did not impact intentions to socially distance, but people’s existing motivations were related to intentions. Findings were generalizable across a geographically diverse sample and may inform public health communication strategies in this and future global health emergencies.

The New Zealand government’s team opted to take a different route, focusing on the impact on people’s daily lives and steps they could take to protect each other … The messaging was overwhelmingly positive in tone, giving “dos” rather than “don’ts” as well as *reasons why*. Instead of “wash your hands,” for instance, the advice was “washing and drying your hands kills the virus”—to underscore individual agency and encourage participation in the national response … In seeking to foster calm and compassion, New Zealand’s messaging was starkly different to that elsewhere. The state of Oregon, for example, ran a campaign with the slogans “Don’t accidentally kill someone” and “It’s up to you how many people live or die.” In the UK, government campaigns have warned “don’t let a coffee cost a life” and shown the reproachful faces of people on ventilators: “Look him in the eyes and tell him the risk isn’t real.” —*The Guardian* (February 22, 2021)

To mitigate the spread of the novel coronavirus (COVID-19) pandemic, international bodies, governments, and other stakeholders around the world have been urging, among other practices, social distancing or maintaining an approximately six foot distance from people who live in other households (1, 2). During the first year of the pandemic, New Zealand emerged as an example of a country that successfully mitigated the spread of COVID-19, which may have been due, in part, to their effective communication strategy (3, 4). Of all the rules that were enforced to various degrees around the world, those that kept people apart from one another, like cancelling public gatherings and restricting movement, were among the most contested, yet effective, interventions to reduce early spread of COVID-19 (5). Longitudinal cross-national studies found that policies like school closures and stay-at-home orders increased social distancing and were effective in slowing COVID-19 daily confirmed cases (6) and deaths (7). Therefore, motivating engagement in social distancing has been emphasized as a critical global public health priority by researchers (8, 9) and global policy makers (1) alike.

Motivation science from the framework of self-determination theory (SDT) can provide insight into why some ways of communicating can motivate behavior change, whereas others, even when well intentioned, may backfire. SDT (10) has long investigated the effects of communication style on the quality, quantity, and sustainability of people’s motivation to change their behavior. New Zealand’s communication strategy as described in the opening quote is one example of an autonomy-supportive communication style that helps people understand and endorse the value of the requested behavior. This communication style involves perspective taking (e.g., acknowledging how difficult it is to alter one’s daily life), providing a meaningful rationale (e.g., explaining why social distancing is effective and important for reducing viral spread), and supporting individual agency and ownership in terms of how to respond within the practical constraints of the situation (e.g., offering safe alternatives from which to choose) (11). In contrast, a controlling communication style, as illustrated with those used by the state of Oregon and the United Kingdom in the opening quote, is characterized by demanding language (e.g., informing people what they should, must or have to do) and relies on shaming and blaming to motivate behavior change (12). Although some argue that controlling messages are necessary in enforcing adherence in the short term (13), this adherence declines over time (14). Moreover, controlling messages can have the opposite effect of increasing undesired behaviors and feelings of defiance or wanting to do the opposite of what is being requested (15, 16). Autonomy-supportive messages, on the other hand, consistently increase adherence in the short and long term (14, 17) and reduce feelings of defiance (15, 16).

Over the course of the COVID-19 pandemic, employers, local governments, national governments, and global health agencies, like the World Health Organization (WHO), have urged people to take various mitigation actions, like social distancing. People have repeatedly defied social distancing recommendations (18, 19); this is not surprising because defiance occurs when people are bombarded with messages to change their behavior and perceive their freedom as restricted (20, 21). This trend of defiance threatens to accelerate viral spread. Thus, establishing whether different messaging approaches can curb feelings of defiance and increase adherence to social distancing recommendations is crucial.

Autonomy-supportive messages about social distancing may be more effective than controlling messages because they promote autonomous motivation or internalizing the value and importance of the requested behavior (e.g., engaging in social distancing to protect their own and others’ health). On the other hand, controlling messages about social distancing may be less effective than autonomy-supportive messages because they promote controlled motivation, a poorly internalized form of motivation relying on avoiding punishment, social judgments, and feelings of shame and guilt (e.g., engaging in social distancing to avoid disapproval from others) (10). Across myriad behaviors, autonomous motivation predicts greater behavior change than controlled motivation in the short and long term (22).

This experiment investigated whether and how communication strategies delivered online in short written messages, a low-cost and common method of conveying public health recommendations (23), could motivate social distancing. Participants recruited from 89 countries were exposed to an autonomy-supportive message, a controlling message, or no message. We recognized that prior to and during the five months of data collection (from April to September 2020), participants were encountering a high volume of messages about social distancing in their everyday lives that varied widely in how autonomy supportive vs. controlling they were. We thus used the “no-message” comparison condition to capture participants’ motivation as a function of exposure to messages received prior to our experiment. Regardless of prior message exposure, we were interested in the magnitude of effects (even if minimal) resulting from exposure to a new motivational message to inform public health stakeholders about realistic effects they could expect to see if implemented at scale.

Three research aims were supported by this design. First, we aimed to determine the extent to which brief written autonomy-supportive and controlling messages differentially affect motivation, feelings of defiance, and behavioral intentions to follow social distancing recommendations. We did not track social distancing adherence over time due to varied resources across the many data collection laboratories and opted to measure behavioral intentions (both short and long term) for social distancing instead. Behavioral intentions or plans to perform a behavior (24) are a key determinant of behavioral adherence and a common outcome for health behavior interventions (24, 25). A second aim was to determine whether the differential effects of autonomy-supportive and controlling messages generalize across a geographically diverse sample (26). Finally, we aimed to test associations between motivations to follow social distancing recommendations and feelings of defiance and behavioral intentions. Recent longitudinal research in Belgium and the United Kingdom suggests that people can simultaneously hold autonomous and controlled motivations for following COVID-19–related recommendations (e.g., handwashing, social distancing, mask wearing) but that only autonomous motivation predicted greater adherence over time; controlled motivation either did not relate or predicted lower adherence over time (27, 28). This global sample allows us to test the generalizability of these differential associations between autonomous and controlled motivation and indicators of adherence to social distancing recommendations, independent of the messaging effects we observe. Finding predictors of defiance and intentions to socially distance that generalize across a global sample, whether from experimental messages or from participants’ existing motivations for social distancing, is critical for informing the best routes of intervention.

Our hypotheses and data analysis plan were preregistered prior to data collection at https://osf.io/2u6xs/.

Hypothesis 1: Compared with the controlling message, those in the autonomy-supportive message and no-message conditions will report 1) higher internalized motivation to socially distance, 2) lower feelings of defiance, and 3) higher short-term (one-week) and long-term (six-month) intentions to socially distance. In other words, we expected the autonomy-supportive message to have benefits over the controlling message and the controlling message to have worse outcomes compared with no message at all.

Hypothesis 2: Autonomous motivation for social distancing will be associated with 1) lower feelings of defiance and greater short-term (one-week) and long-term (six-month) intentions to socially distance, while controlled motivation will 2) have inverse associations with defiance and behavioral intentions.

## Results

### Descriptive Statistics.

**Fig. 1.**
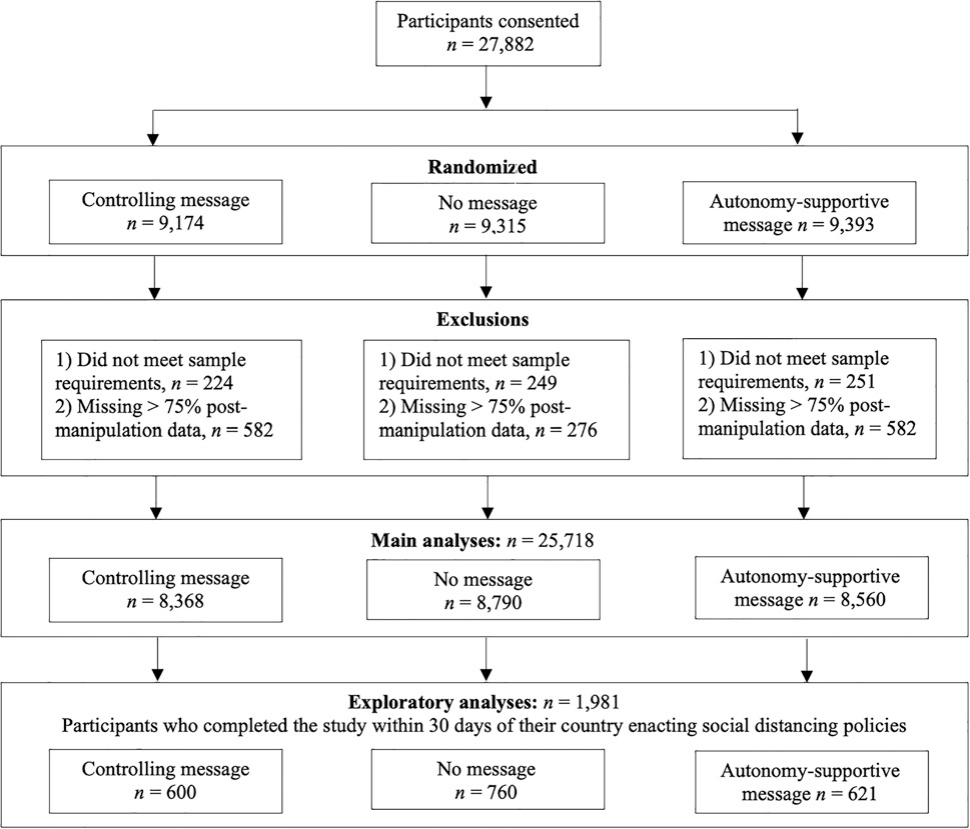
Flowchart delineating the final samples used in analyses.

**Fig. 2.**
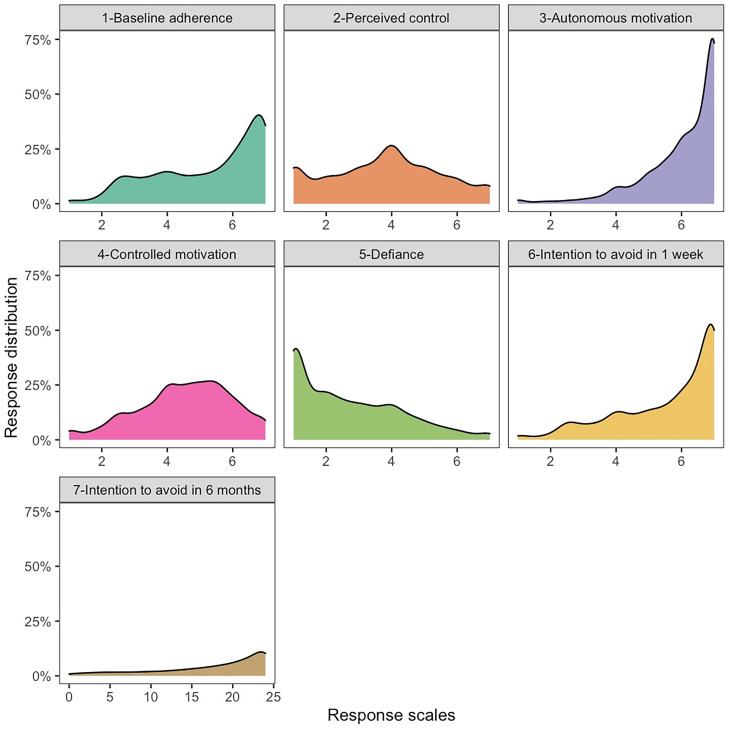
Data distributions for all study variables (the *y* axis indicates the proportion of sample, and the *x* axis indicates response scales).

Descriptive statistics for all variables analyzed in this study, including correlations among variables, are presented in Table 1. Fig. 1 shows the final samples used in analyses after data exclusions (*SI Appendix* has a description). Fig. 2 shows distributions of study variables, indicating that, on average, participants were already following social distancing to a high degree, they intended to continue following recommendations in the future, they already highly endorsed the value of the recommendations, and they reported feeling very little defiance about these recommendations.

**Table 1.** Reliabilities, means, SDs, and correlations with CIs

Variable	*α*/*ω*	ICC	M (SD)	Condition M (SD)			1	2	3	4	5	6
				C	NM	AS						
1) Baseline adherence	0.88/0.91	0.15	5.24	5.22	5.26	5.23						
			(1.60)	(1.62)	(1.60)	(1.59)						
2) Perceived control*	0.67/0.67	0.04	3.79	4.15	3.76	3.46	–0.13**					
			(1.72)	(1.78)	(1.67)	(1.63)	[–0.14, –0.12]					
3) Autonomous motivation	0.96/0.97	0.14	6.02	6.01	5.96	6.09	0.38**	–0.35**				
			(1.18)	(1.21)	(1.22)	(1.10)	[0.37, 0.39]	[–0.36, –0.34]				
4) Controlled motivation	0.71/0.77	0.10	4.53	4.68	4.34	4.58	0.10**	0.11**	0.28**			
			(1.42)	(1.42)	(1.45)	(1.38)	[0.09, 0.11]	[0.10, 0.12]	[0.27, 0.29]			
5) Defiance	0.91/0.93	0.05	2.71	2.79	2.79	2.54	–0.22**	0.52**	–0.47**	0.04**		
			(1.60)	(1.68)	(1.58)	(1.53)	[–0.24, –0.21]	[0.51, 0.53]	[–0.48, –0.47]	[0.03, 0.05]		
6) Intention to social distance next 1 wk	0.91/0.93	0.13	5.57	5.54	5.60	5.56	0.57**	–0.16**	0.46**	0.14**	–0.28**	
			(1.53)	(1.54)	(1.53)	(1.52)	[0.57, 0.58]	[–0.17, –0.15]	[0.450.47]	[0.13, 0.16]	[–0.29, –0.26]	
7) Intention to social distance next 6 mo^†^	0.90/0.92	0.09	17.51	17.61	17.56	17.37	0.39**	–0.28**	0.47**	0.05**	–0.41**	0.43**
			(6.74)	(6.77)	(6.68)	(6.79)	[0.380.40]	[–0.30, –0.27]	[0.460.48]	[0.03, 0.06]	[–0.42, –0.40]	[0.42, 0.44]

*N* = 25,718. M and SD are used to represent mean and SD, respectively. ICC is the intraclass correlation coefficient. Values in square brackets indicate the 95% CI for each correlation. The 95% CI is a plausible range of population correlations that could have caused the sample correlation. AS, autonomy supportive; C, controlling; NM, no message. **P < 0.001.

*Only two items were included for this variable: “try to pressure people” and “aren’t very sensitive to people’s needs.” The original three-item measure yielded *α* = 0.55 and *ω* = 0.62. We preregistered that if *α* or *ω* < 0.70, the composite would only include items with corrected item–total correlations above 0.30. More details are in *SI Appendix*.

^†^Excluding erroneous data.

### Confirmatory Analyses.

Given the large sample size in this study, confirmatory analyses were preregistered with a specified region of practical equivalence to aid interpretation of statistically significant but small effects. We specified that a hypothesis would be supported if an effect and its 95% CI were fully outside of the null interval of *d* = −0.050 to 0.050 (equivalent to partial *r* [*r_p_*] = −0.025 to 0.025). If an effect and its 95% CI overlap with the null interval, it would not be considered practically meaningful, and the hypothesis would not be supported. This cutoff was informed by *d* = |0.05| as our smallest effect size of interest.*

Results reported in the text focus on partial *r* (*r_p_*) for random intercept models (Table 2 shows a more complete reporting of the statistics, and Table 3 presents these models adding in random slopes for predictor variables). *SI Appendix* has additional analyses.^†^

**Table 2.** Random intercept–only models testing confirmatory effects of experimental conditions (Hypothesis 1) and autonomous and controlled motivation (Hypothesis 2) on outcomes

Outcome and term	*B*	SE	*t*	df	*r_p_*	95% CI around *r_p_*		*P* value	Variance of random effects
						Lower	Upper		
Autonomous motivation Hypothesis 1									
Controlling (intercept)	6.01	0.06	107.99	76.01	0.048	0.036	0.060	<0.001	0.191
Vs. no message	−0.04	0.02	−2.10	25,649.85	–0.012	–0.024	-0.001	0.036	
Vs. autonomy supportive	0.10	0.02	5.83	25,649.03	0.034	0.021	0.046	<0.001	
Controlled motivation Hypothesis 1									
Controlling (intercept)	4.57	0.06	78.37	77.52	0.099	0.088	0.112	<0.001	0.20
Vs. no message	−0.34	0.02	−16.24	25,646.41	–0.096	–0.108	–0.084	<0.001	
Vs. autonomy supportive	−0.09	0.02	−4.47	25,644.91	–0.026	–0.039	–0.014	<0.001	
Defiance Hypothesis 1									
Controlling (intercept)	2.77	0.05	55.54	69.88	0.073	0.061	0.085	<0.001	0.13
Vs. no message	−0.01	0.02	−0.44	25,412.46	–0.003	–0.015	0.000	0.657	
Vs. autonomy supportive	−0.25	0.02	−10.50	25,409.08	–0.064	–0.076	–0.052	<0.001	
Defiance Hypothesis 2									
Intercept	6.20	0.07	93.79	297.72	0.524	0.516	0.532	<0.001	0.11
Autonomous motivation	−0.75	0.01	−94.64	25,338.34	–0.522	–0.530	–0.514	<0.001	
Controlled motivation	0.23	0.01	36.10	25,413.67	0.223	0.211	0.234	<0.001	
Intention to avoid 1 wk Hypothesis 1									
Controlling (intercept)	5.42	0.07	77.26	74.77	0.017	0.007	0.030	<0.001	0.30
Vs. no message	0.06	0.02	2.91	25,235.70	0.017	0.005	0.029	0.004	
Vs. autonomy supportive	0.03	0.02	1.52	25,234.29	0.009	0.001	0.021	0.128	
Intention to avoid 1 wk Hypothesis 2									
Intercept	2.00	0.07	28.24	212.79	0.446	0.437	0.456	<0.001	0.17
Autonomous motivation	0.58	0.01	75.29	25,252.95	0.433	0.423	0.442	<0.001	
Controlled motivation	−0.01	0.01	−0.92	25,265.99	–0.006	–0.018	0.000	0.355	
Intention to avoid 6 mo Hypothesis 1*									
Controlling (intercept)	17.20	0.27	64.42	72.23	0.012	0.003	0.025	<0.001	4.02
Vs. no message	−0.01	0.10	−0.10	24,606.22	–0.001	–0.014	0.000	0.917	
Vs. autonomy supportive	−0.17	0.10	−1.72	24,604.00	–0.010	–0.023	–0.001	0.086	
Intention to avoid 6 mo Hypothesis 2*									
Intercept	2.50	0.29	8.75	292.37	0.466	0.457	0.475	<0.001	2.05
Autonomous motivation	2.76	0.03	79.95	24,528.81	0.465	0.456	0.474	<0.001	
Controlled motivation	−0.45	0.03	−15.97	24,607.37	–0.102	–0.114	–0.090	<0.001	

*B* is the unstandardized coefficient; *r_p_* is the partial standardized effect size for each coefficient. *N* = 25,718. Controlling: *n* = 8,368; no message: *n* = 8,790; autonomy supportive: *n* = 8,560. The controlling message was the reference group. We report three decimal places for *p* and *r_p_* and its 95% CI since our interval null is *r_p_* = –0.025 to 0.025 and two decimals for all other values. df, degree of freedom.

^*^Excluding erroneous data.

**Table 3.** Maximal models testing the confirmatory effect of experimental conditions (Hypothesis 1) and autonomous and controlled motivation (Hypothesis 2) on outcomes only using countries with a sample size of 210 or above

Outcome and term	*B*	SE	*t*	df	*r_p_*	95% CI around *r_p_*		*P* value	Variance of random effects
						Lower	Upper		
Autonomous motivation Hypothesis 1									
Controlling (intercept)	5.99	0.08	73.82	34.81	0.046	0.034	0.059	<0.001	0.22
Vs. no message	−0.03	0.03	−1.25	23.72	–0.012	–0.024	–0.001	0.223	0.01
Vs. autonomy supportive	0.10	0.03	3.48	26.33	0.033	0.020	0.045	0.002	0.01
Controlled motivation Hypothesis 1									
Controlling (intercept)	4.66	0.08	59.42	34.51	0.097	0.085	0.110	<0.001	0.20
Vs. no message	−0.33	0.03	−11.98	19.91	–0.094	–0.107	–0.082	<0.001	0.01
Vs. autonomy supportive	−0.10	0.02	−3.91	24.50	–0.027	–0.040	–0.014	0.001	0.00
Defiance Hypothesis 1									
Controlling (intercept)	2.79	0.06	46.36	32.80	0.064	0.051	0.077	<0.001	0.11
Vs. no message	−0.04	0.06	−0.70	33.18	–0.011	–0.024	–0.001	0.487	0.10
Vs. autonomy supportive	−0.24	0.06	−3.68	33.49	–0.060	–0.073	–0.047	0.001	0.11
Defiance Hypothesis 2									
Intercept	5.98	0.18	32.68	31.95	0.518	0.510	0.527	<0.001	1.00
Autonomous motivation	−0.74	0.03	−24.54	34.19	–0.515	–0.524	–0.506	<0.001	0.03
Controlled motivation	0.26	0.02	11.45	31.53	0.244	0.232	0.255	<0.001	0.01
Intention to avoid 1 wk Hypothesis 1									
Controlling (intercept)	5.37	0.09	60.85	34.63	0.016	0.005	0.030	<0.001	0.26
Vs. no message	0.06	0.03	1.82	20.53	0.015	0.002	0.028	0.083	0.01
Vs. autonomy supportive	0.05	0.02	1.96	779.67	0.012	0.001	0.025	0.050	0.00
Intention to avoid 1 wk Hypothesis 2									
Intercept	2.20	0.21	10.70	34.64	0.425	0.415	0.435	<0.001	1.32
Autonomous motivation	0.54	0.03	16.35	34.93	0.413	0.402	0.423	<0.001	0.03
Controlled motivation	−0.01	0.01	−1.36	13.98	–0.010	–0.023	–0.001	0.196	0.00
Intention to avoid 6 mo Hypothesis 1*									
Controlling (intercept)	17.27	0.33	52.70	35.59	0.008	0.002	0.023	<0.001	3.44
Vs. no message	0.07	0.12	0.57	22.48	0.004	0.000	0.017	0.573	0.08
Vs. autonomy supportive	−0.06	0.14	−0.43	17.49	–0.004	–0.017	-0.000	0.671	0.20
Intention to avoid 6 mo Hypothesis 2*									
Intercept	3.32	0.82	4.07	30.70	0.453	0.443	0.463	<0.001	19.68
Autonomous motivation	2.68	0.13	20.79	30.27	0.452	0.442	0.462	<0.001	0.49
Controlled motivation	−0.48	0.07	−6.59	23.72	–0.108	–0.121	–0.095	<0.001	0.14

*B* is the unstandardized coefficient; *r_p_* is the partial standardized effect size for each coefficient. *N* = 23,554. Controlling: *n* = 7,688; no message: *n* = 8,059; autonomy supportive: *n* = 7,807. The controlling message was the reference group. We report three decimal places for *p* and *r_p_* and its 95% CI since our interval null is *r_p_* = –0.025 to 0.025 and two decimals for all other values. df, degree of freedom.

*Excluding erroneous data.

#### Hypothesis 1.

**Fig. 3.**
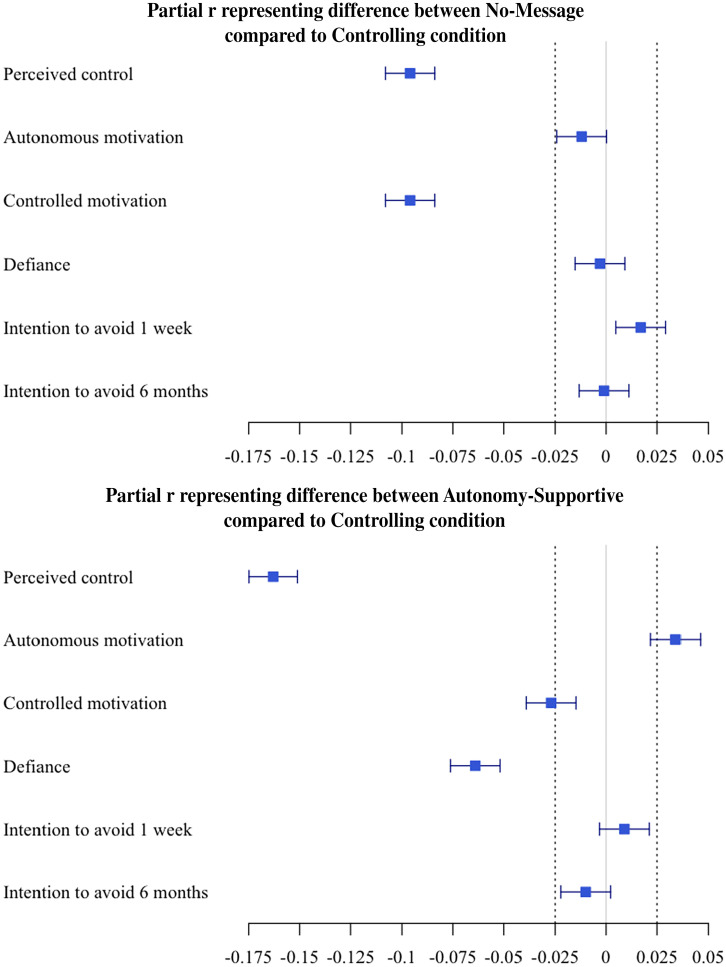
Illustrating confirmatory effects testing Hypothesis 1. Effect sizes are drawn from intercept-only models in Table 2 (*n* = 25,718). Values to the left of zero indicate that no message (or the autonomy-supportive message) yielded lower scores on outcomes than the controlling message. Values to the right of zero indicate that no message (or the autonomy-supportive message) yielded higher scores on those outcomes than the controlling message. The square represents the observed effect size, and the whiskers represents the 95% CIs; if the effect and its 95% CI fall outside the dotted lines (the interval null of *r_p_* = −0.025 to 0.025), the effect is considered practically meaningful.

Fig. 3 shows a visualization of confirmatory effects for Hypothesis 1.

##### Autonomous and controlled motivation.

Across all message conditions, autonomous motivation was high (mean and SD in Table 1). We did not find evidence that the autonomy-supportive message condition meaningfully yielded higher autonomous motivation than the controlling message condition (*r_p_* = 0.034, 95% CI [0.021, 0.046]), nor did we find evidence that those in the no-message condition reported higher autonomous motivation than those in the controlling message condition (*r_p_* = –0.012, 95% CI [–0.024, 0.001]).

Across all message conditions, controlled motivation was moderate. Those in the no-message condition showed lower controlled motivation than those in the controlling message condition (*r_p_* = –0.096, 95% CI [–0.108, –0.084]). However, we did not find evidence of a difference in controlled motivation between the autonomy-supportive message and controlling message conditions (*r_p_* = –0.026, 95% CI [–0.039, –0.014]).

##### Feelings of defiance.

Across conditions, feelings of defiance were low. The autonomy-supportive message led to lower feelings of defiance than the controlling message (*r_p_* = –0.064, 95% CI [–0.076, –0.052]). However, we did not find a difference between the no-message and the controlling message conditions (*r_p_* = –0.003, 95% CI [–0.015, 0.000]).

##### Short- and long-term behavioral intentions.

People generally intended to socially distance in the next week and intended to continue socially distancing for the majority of the next six months. The autonomy-supportive message condition did not yield differences in one-week social distancing intentions from the controlling message condition (*r_p_* = 0.009, 95% CI [0.001, 0.021]), nor did the no-message condition (*r_p_* = 0.017, 95% CI [0.005, 0.029]). Similarly, the autonomy-supportive message condition did not yield differences in social distancing intentions in the next six months from the controlling message condition (*r_p_* = –0.010, 95% CI [–0.023, –0.001]), nor did the no-message condition (*r_p_* = –0.001, 95% CI [–0.014, 0.000]). Thus, we did not find that conditions differed in short- or long-term behavioral intentions to socially distance.

#### Hypothesis 2.

##### Feelings of defiance.

As expected, autonomous motivation predicted lower feelings of defiance (*r_p_* = –0.522, 95% CI [–0.530, –0.514]). Additionally, controlled motivation predicted higher feelings of defiance (*r_p_* = 0.223, 95% CI [0.211, 0.234]).

##### Short- and longer-term behavioral intentions.

Autonomous motivation was associated with greater intentions to socially distance in the next week (*r_p_* = 0.433, 95% CI [0.423, 0.442]), whereas controlled motivation was not related to short-term behavioral intentions (*r_p_* = –0.006, 95% CI [–0.018, 0.000]). Autonomous motivation was positively associated with behavioral intentions to socially distance in the next six months (*r_p_* = 0.465, 95% CI [0.456, 0.474]), whereas controlled motivation was negatively associated with longer-term behavioral intentions (*r_p_* = –0.102, 95% CI [–0.114, –0.090]).

### Exploratory Analyses.

We conducted exploratory analyses using the same analytical approach to test our hypotheses on a subsample of participants who took the study within the first month of their country enacting lockdowns and other policies enabling social distancing (*n* = 1,981) (Table 4).^‡^ The rationale for this analysis was to examine whether the effects of our manipulation might be larger early on in the pandemic. In this analysis, we also included a covariate—country’s total cases per million—to test for the possibility that the country-specific incidence rate may predict motivation, feelings of defiance, and behavioral intentions. Results showed evidence for two additional experimental effects: the autonomy-supportive message increased autonomous motivation to engage in social distancing relative to the controlling message (*r_p_* = 0.117, 95% CI [0.073, 0.160]), and the controlling message increased feelings of defiance relative to no message, *r_p_* = -0.130, 95% CI [-0.173, -0.087]. We also observed a larger effect of the autonomy-suppportive message eliciting lower feelings of defiance than the controlling message in this subsample, *r_p_* = -0.217, 95% CI [-0.258, -0.175]. The effect of the controlling message increasing controlled motivation to engage in social distancing relative to no message remained (*r_p_* = –0.107, 95% CI [–0.151, –0.064]). We also observed a larger effect of the autonomy-supportive message eliciting lower feelings of defiance than the controlling message in this subsample (*r_p_* = –0.217, 95% CI [–0.258, –0.175]). Just as in the full sample, we did not find evidence of a difference between the controlling and no-message conditions on defiance, nor did we find condition differences on short- or long-term behavioral intentions. With respect to our exploratory covariate, we found that country-specific incidence rate correlated with greater intentions for social distancing in the next six months (*r_p_* = 0.445, 95% CI [0.410, 0.479]).

**Table 4.** Random intercept-only models testing Hypothesis 1: the effects of condition on outcome variables for the sample of participants who completed surveys within 30 days since their country’s rise in restrictions

	*B*	SE	*t*	df	*r_p_*	95% CI around *r_p_*		*P* value	Variance of random effects
						Lower	Upper		
Autonomous motivation									
Controlling (intercept)	6.35	0.07	92.55	3.07	0.120	0.082	0.167	<0.001	0.00
Vs. no message	0.07	0.04	1.68	1,976.40	0.038	0.003	0.081	0.094	
Vs. autonomy supportive	0.24	0.05	5.24	1,980.63	0.117	0.073	0.160	<0.001	
Covariate: Total cases per million	−2.78E-06	4.26E-05	−0.07	2.39	-0.003	-0.051	-0.001	0.953	
Controlled motivation									
Controlling (intercept)	4.97	0.24	20.91	5.43	0.123	0.085	0.170	<0.001	0.07
Vs. no message	−0.36	0.07	−4.89	1,976.52	–0.107	–0.151	–0.064	<0.001	
Vs. autonomy supportive	−0.23	0.08	−2.92	1,977.58	–0.064	–0.108	–0.021	0.004	
Covariate: Total cases per million	1.01E-04	1.49E-04	0.68	6.97	0.064	0.020	0.108	0.519	
Defiance									
Controlling (intercept)	2.66	0.09	29.17	2.23	0.227	0.188	0.270	0.001	0.00
Vs. no message	−0.42	0.07	−5.83	1,955.26	–0.130	–0.173	–0.087	<0.001	
Vs. autonomy supportive	−0.74	0.07	−9.84	1,960.96	–0.217	–0.258	–0.175	<0.001	
Covariate: Total cases per million	1.10E-04	5.49E-05	2.01	1.51	0.074	0.030	0.118	0.222	
Intention to avoid next 1 wk									
Controlling (intercept)	6.44	0.06	104.98	0.94	0.070	0.037	0.120	0.008	0.00
Vs. no message	0.04	0.06	0.75	1,929.64	0.017	0.001	0.062	0.451	
Vs. autonomy supportive	0.10	0.06	1.66	1,943.00	0.038	0.003	0.082	0.097	
Covariate: Total cases per million	7.05E-05	3.49E-05	2.02	0.52	0.059	0.015	0.103	0.433	
Intention to avoid next 6 mo									
Controlling (intercept)	15.71	2.07	7.59	5.91	0.445	0.411	0.479	<0.001	14.32
Vs. no message	−0.38	0.22	−1.75	1,893.51	–0.029	–0.074	–0.002	0.080	
Vs. autonomy supportive	−0.25	0.23	−1.10	1,892.62	–0.018	–0.063	–0.001	0.273	
Covariate: Total cases per million	3.08E-03	8.80E-04	3.50	45.63	0.445	0.410	0.479	0.001	

*B* is the unstandardized coefficient; *r_p_* is the partial standardized effect size for each coefficient. *N* = 1,981. Controlling: *n* = 600; no message: *n* = 760; autonomy supportive: *n* = 621. The controlling message was the reference group. We report three decimal places for *p* and *r_p_* and its 95% CI since our interval null is *r_p_* = –0.025 to 0.025 and two decimals for all other values. df, degree of freedom.

## Discussion

Public health communications play a critical role in managing health emergencies, including during pandemics, by motivating people to engage in behaviors like handwashing, mask wearing, vaccine uptake, and social distancing (26). Here, we tested motivational qualities (autonomy supportive vs. controlling) of messages about social distancing in individuals recruited across 89 countries. The aim was to identify empirically supported communication strategies that can be generalized cross-culturally to inform public health practices not only in this but also, in future global health emergencies.

We found evidence for two confirmatory experimental effects. 1) The controlling, pressuring message increased controlled motivation to follow recommendations out of guilt and fear of social punishment more than the messages to which participants had been previously exposed. 2) The autonomy-supportive message that promoted agency and ownership lowered feelings of defiance relative to the controlling message. Furthermore, exploratory analyses focusing on message delivery early on in the pandemic (i.e., within the first month after countries instituted lockdowns and other policies urging social distancing) found two more effects; compared with the controlling message, the autonomy-supportive message increased autonomous motivation or internalizing the value of social distancing, and the controlling message increased feelings of defiance relative to no message at all. The confirmatory experimental effects are small according to Cohen’s benchmarks (29), but they were in line with effect sizes observed in a meta-analysis of health messaging campaigns (average *r* = 0.09, 95% CI [0.07, 0.10], *r* values ranging from 0.04 to 0.15). Notably, this meta-analysis (30) found that effects tend to be smaller for media campaigns motivating avoidance behaviors (e.g., the average effect size for smoking cessation media campaigns was *r* = 0.03, 95% CI [0.02, 0.04]), which could explain the small effect sizes we found when motivating people to avoid gathering with others.

However, we did not find evidence for effects of either autonomy-supportive or controlling messages on short- or long-term intentions to follow social distancing recommendations. We consider several possibilities that may contribute to the lack of messaging effects on behavioral intentions. First, it could be due to a ceiling effect of adherence to social distancing recommendations, making it difficult to increase adherence that is already very high. Second, by the time data collection started in mid-April 2020, participants had already been exposed to hundreds, if not thousands, of messages promoting social distancing with varying motivational content. As a result, the potential impact of a single message on people’s short-term and long-term intentions to engage in social distancing may be negligible relative to a context where participants were exposed to a new health message for the first and potentially, only time. As well, the “dosage” of our intervention—one brief (two-minute) written message—is likely less effective than receiving autonomy support during an intervention that might last weeks or months (17). Asking people to alter their daily lives to abstain from social interactions might require more time and effort than the brief online message we provided. Finally, there may be complex factors preventing social distancing (e.g., maintaining one’s livelihood, traveling to care for sick relatives) that may require tangible, economic interventions before messages can have an impact (31).

Compared with the experimental effects of motivational messages, people’s existing motivations for social distancing were better predictors of behavioral intentions, fully supporting Hypothesis 2. In particular, those who reported higher motivation driven by the value and importance of social distancing expressed greater behavioral intentions to engage in social distancing in both the short term and long term. Conversely, following social distancing rules out of guilt and fear of social punishment correlated with lower long-term behavioral intentions. Further, exploratory analyses focused on the first wave of the pandemic found that higher daily cases were associated with greater long-term intentions to socially distance.

Taken together, results suggest that intentions to adhere to social distancing recommendations were explained more by people’s existing motivations and perceptions of viral risk than the messages used in this study. From these data, we can conclude only that autonomy-supportive vs. controlling aspects of messages urging social distancing mattered in terms of affecting public sentiments toward social distancing (e.g., increasing feelings of defiance) but not people’s intention to carry it out. Even so, public sentiment plays a key role in supporting public health measures and in the effectiveness of managing health emergencies (32, 33).

### Design Limitations and Future Directions.

First, due to convenience sampling methods, distributions of study variables suggest that our sample was highly autonomously motivated, already engaged in social distancing, and had very low feelings of defiance. Therefore, our results may not be generalizable to those who might have resisted social distancing or those who lived in areas where social distancing rules were not imposed. Additionally, we did not investigate whether message type (autonomy supportive or controlling) might be more or less effective in influencing outcomes as a function of its source/communicator (e.g., expertise, trustworthiness) (34, 35), cultural context (e.g., individualistic–collectivistic, democratic–authoritarian, cultural tightness–looseness, interpersonal distance preferences) (36–38), local or national infection rates, or legal restrictions (6). For example, a recent study by Gelfand et al. (37) suggests that countries that score higher on cultural tightness show lower death rates compared with countries with looser cultures, which tend to be less strict about norm deviance. As such, it seems plausible that cultural tightness vs. looseness may impact how motivational messages are interpreted, and this should be investigated in future work. Although the current study aimed to identify generalizable benefits and harms of different motivational communication styles, we encourage researchers to use this dataset and the larger Psychological Science Accelerator COVID-19 Rapid Project (PSACR) dataset (https://osf.io/gvw56/) to examine these and other questions.

### Conclusions.

We conclude that in a public health context, autonomy-supportive messages have some benefits over controlling messages for motivation and feelings of defiance (although we did not find evidence that messages mattered for people’s behavioral intentions). Messaging effects on motivation and feelings of defiance observed in this study were small, but they likely have meaningful real-world impacts when accumulated across time and global populations (39, 40), whereas their effects on intentions to comply with social distancing recommendations likely do not. The strength of the manipulation used in this study is the ease and efficiency of producing and digitally disseminating these brief messages that can reach a large number of people in a short amount of time. Findings may have similar applications for other public health behavioral recommendations, including mask wearing, handwashing, self-quarantining after exposure, and vaccination, for which evidence of defiance has also been observed (41). Readers seeking further guidance for applying SDT to motivate COVID-19–related behavioral recommendations may also review Martela et al. (42) and Bradshaw et al. (43). Finally, while SDT principles for strategic communication likely apply to motivating other behaviors of interest to public health stakeholders, communications aimed at modifying behavior should be evaluated on many dimensions, including ease of implementation and sustainability of impacts, such as with the reach, effectiveness, adoption, implementation, and maintenance framework (44).

This study represents a major undertaking and truly international collaboration involving the coordination of laboratories in 89 different countries and collecting a total sample of 25,718 participants. The strongest findings from this research support the generalizability of meaningful and differential relations between people’s existing motivations on public health compliance intentions, suggesting benefits of cultivating autonomous motivation and limiting controlled motivation. The effects of messages were more modest; the controlling message increased feelings of defiance relative to the autonomy-supportive message and increased controlled motivation—a less optimal form of motivation associated with lower intentions to socially distance—relative to no message. This research, including the cross-national sample and transparent reporting of materials and data (https://osf.io/fc9y7/), can help advance future research and applications of evidence-based health communication on a global scale for the current COVID-19 pandemic and for future public health crises.

## Materials and Methods

This study was one of three studies in the PSACR (https://psyarxiv.com/x976j/ has details about logistics and additional measures administered). Through the Psychological Science Accelerator (PSA) (45), the methodological approach, measures, and analytic strategy received extensive feedback from coauthors and external reviewers before data collection began.

### Participants.

Through the PSACR project, data were collected from ∼186 laboratories^§^ across 87 autonomous regions and countries (PSA network laboratories). Data from 26 laboratories across 17 countries (with 2 nonoverlapping countries) were collected from SDT network laboratories (invited through the SDT listserv).^¶^ Participating laboratories recruited participants via local university subject pools or relied on social media posts and emails to invite those in their personal networks to participate. Additionally, our sample also included 5,304 additional participants recruited through semirepresentative panels (quota matched to the general population in terms of sex and age) from the following countries: Austria, China, Egypt, Japan, Kenya, Mexico, Nigeria, Romania, Russia, South Africa, South Korea, Sweden, Switzerland, Thailand, Turkey, the United Kingdom, and the United States (with ∼270 participants per country on average). Participants’ compensation differed depending on how they were recruited and which laboratory recruited them. As such, some participants received payments, others received course credit at their university, and some did not receive compensation (more details on recruitment and compensation are at https://psyarxiv.com/x976j/).

After data exclusions (Fig. 1), our final sample was 25,718 participants across 89 countries, representing all inhabited continents. *SI Appendix*, Table S1 shows a list of sample sizes corresponding to each country. Of the total sample, 63.3% identified as female (*n* = 16,273), 33.6% identified as male (*n* = 8,636), 1.1% indicated that male and female categories did not fit for them (*n* = 288), and 2% preferred not to respond. The age of the sample ranged between 18 and 89 years, with a mean age of 37 years (SD = 15.6).

### Experimental Manipulation.

Participants were randomly assigned to an autonomy-supportive message condition, a controlling message condition, or a no-message condition. The autonomy-supportive and controlling message conditions presented comparable information about social distancing, including its definition, its implications for public health during the COVID-19 outbreak, and neutral informative behavioral recommendations. Alongside this basic content, both messages contained theory-based motivational elements shown in prior manipulations to influence motivation (15, 46). Specifically, those in the autonomy-supportive message condition read an article that provided 1) perspective taking (e.g., acknowledging how difficult it is to alter one’s daily life), 2) a meaningful rationale (e.g., explaining why social distancing is effective and important for slowing transmission), and 3) a sense of having choice over one’s own behavior within the practical constraints of the situation. In comparison, those in the controlling message condition read an article that paired information with coercion, shame, and pressure, including the use of demanding language, such as “should” and “must.” Finally, those in the no-message condition did not read any message; instead, they directly responded to the outcome measures.

### Measures.

For all multiitem measures, items were reverse scored where appropriate and then, combined into composites for our variables. Per the preregistration, if a composite variable did not have acceptable reliability (*ω_total_* > 0.70), we retained items with corrected item–total correlations exceeding 0.30 (Table 1). The wording of outcome items differed slightly depending on condition. In the autonomy-supportive and controlling message conditions, items referred to “social distancing recommendations in this article,” while in the no-message condition, items referred to “social distancing recommendations” (not tied to an article).

#### Autonomous and controlled motivation.

Following random assignment to see an autonomy-supportive message, a controlling message, or no message, participants completed a measure of their motivation to follow social distancing recommendations. This measure was adapted from a previous measure of perceived locus of causality (47, 48) for the behavior of social distancing. Participants responded to the prompt “I plan to follow social distancing recommendations [in this article] because” with four autonomous and four controlled reasons for doing so. Example items assessing autonomous motivation included “the recommendations reflect my values” and “it is personally important to me to follow them.” Example items assessing controlled motivation included “because others would disapprove of me if I did not” and “I would feel guilty if I did not follow the recommendation.” The items were paired with a seven-point scale (1 = strongly disagree, 7 = strongly agree). Autonomous and controlled motivation items were aggregated into two separate variables for analyses as both scales showed good reliability (autonomous motivation: *ω* = 0.90; controlled motivation: *ω* = 0.77).

#### Feelings of defiance.

Feelings of defiance were measured with four items adapted from Vansteenkiste et al. (49). Items measured feelings of defiance about “recommendations [in this article] on social distancing, or staying home as much as possible” and were rated on a seven-point scale (1 = strongly disagree, 7 = strongly agree). The items were “make me feel like I want to do exactly the opposite,” “feel aggravating,” “feel like an intrusion,” and “make me want to resist attempts to influence me.” These items showed good reliability (*ω* = 0.89).

#### Short- and long-term behavioral intentions.

Intentions were measured at a more abstract level of actions (e.g., “following recommendations to participate in social distancing”) as well as at a lower and more concrete level of actions (e.g., “avoid gatherings with friends”) as both contribute to goal pursuit [reviewed by Freund and Hennecke (50)]. Our behavioral intention items were adapted from Armitage and Conner (51) and Flannelly et al. (52), following an adaptation by McGarrity and Huebner (53), to assess participants’ intentions for social distancing. Items assessing short-term intentions asked participants how likely they would be to “follow the recommendation to participate in social distancing” and avoid “gatherings with friends,” “going to crowded areas,” and “taking nonessential shopping trips” in the next week. The response scale ranged from 1 = extremely unlikely to 7 = extremely likely. The scale showed good reliability for all four items combined (*ω* = 0.88). The measure for long-term intentions asked, “assuming the guidelines [described in the article] last for six months, how long do you intend on avoiding the following in-person places and activities,” and the list of activities included “restaurants,” “gatherings with friends,” “traveling,” “going in crowded areas,” “nonessential shopping trips,” “getting a haircut or going to the salon,” and “going to the gym or fitness classes.” These items were rated in one-week increments using a drop-down menu from 0 to 24 weeks. An average score was calculated for all seven items as they showed good reliability (*ω* = 0.92).

#### Demographic information.

Demographics assessed by both PSA and SDT laboratories were age, gender, education, and country. The PSACR general survey (https://osf.io/ecba8/) also collected additional demographic and background variables related to COVID-19 beyond the scope of this study.

### Design and Procedure.

All data collection laboratories followed the ethical guidelines of their institutions. Guidelines for internet-based data collection were followed where applicable (54). Each laboratory 1) received ethical approval from their local institutional review board (IRB), 2) gained approval through Ashland University’s Human Subject’s Review Board (for the PSA laboratories) or through the Illinois Institute of Technology’s IRB (for the SDT laboratories), or 3) did not require local IRB approval for data collection. All participants provided informed consent before entering the study.

Participants completed the study online between mid-April 2020 and the end of September 2020. Data were collected using formr (55) for PSACR laboratories and Qualtrics for SDT laboratories. Some participants completed our study along with another PSACR experiment in random order; order was recorded to examine potential carryover effects. More information about study design, translations, and measures of baseline social distancing adherence and perceived control used for the manipulation check is in *SI Appendix*.

### Analytic Plan.

#### Modeling approach.

All analyses were conducted in R (version 1.3.1056). To account for the nested structure of the data, we used mixed effects models in the statistical package lme4 (version 1.1-21) (56). In testing Hypothesis 1, the controlling message condition served as the reference group and was compared with the autonomy-supportive and no-message conditions. For Hypothesis 2, controlled and autonomous motivation were entered as simultaneous predictors.

We focus on random intercept models in the text. We estimated models with and without random slopes, with nearly identical results (Tables 2 and 3). The equation of the random intercept models is as follows:

$$Y_{iC}=\;\beta_{0}+\beta_{1}\cdot \mathrm{NoMessag}e_{iC}+\beta_{2}\cdot Au\mathrm{tonomySupportiv}e_{iC}+\;u_{0C}+\;e_{iC}.$$

In this equation, each observation is clustered within grouping variable *c* (country).

$\beta_{0}$ is the overall intercept for the reference group (the controlling message condition), and $u_{0C}$ is the random effect of the intercept. The fixed effects include $\beta_{1}$ and $\beta_{2}$, which are the slopes representing the difference between the no-message condition and the autonomy-supportive message condition, respectively, and the controlling message condition.

We used the TOSTER package (version 0.3.4) (57) to illustrate fixed effects and their 95% CIs (Fig. 3) and calculated partial *r* (*r_p_*) values (standardized effect sizes) using the r2beta function in r2glmm (version 0.1.2) (58).

#### Exploratory analyses.

Data collection launched in April 2020 and continued through September 2020. We speculated that communication strategies urging social distancing might have been more impactful early on in our data collection period before message fatigue or exhaustion from prolonged exposure to social distancing messages, set in (21). Thus, we explored message effects among those who completed the study within 30 days of their country first enacting policies aimed at promoting social distancing. To identify those participants, we used the publicly available dataset Our World in Data (59). From this dataset, we extracted two types of information. First, we extracted stringency index data from the Oxford COVID-19 Government Response Tracker (60) to identify when there was the steepest increase in lockdowns and other policies aimed at social distancing (e.g., school closures) within two consecutive weeks. This happened in March and early April for all countries available in our sample. We restricted the sample in exploratory analyses to those who completed the study within the first 30 days after their country’s rise in these policies. Second, we extracted data that came from the COVID-19 Data Repository by the Center for Systems Science and Engineering at Johns Hopkins University (61) on the incidence rate in a country (cases per million to account for population differences) at the time participants completed the study. We defined country-specific incidence rate as a covariate in exploratory analyses, allowing us to test the possibility that motivation, feelings of defiance, and behavioral intentions to socially distance would be predicted by case numbers in that country. Together, this analytic approach provided a more sensitive test of a country’s unique pandemic experience during its first wave. Because some countries had small amounts of data during this early time period, we only included random intercepts but not random slopes for these analyses.

## Supplementary Material

Supplementary File

## Data Availability

The preregistration, materials, analytic plan, code, and data to reproduce all analyses have been deposited in the Open Science Framework (62).

## Footnotes

^*^We deviated from our preregistration in that we report partial *r* (*r_p_*) instead of Cohen’s *d* because our planned analyses produced *r_p_*; reporting *r_p_* also makes results easier to compare with previous findings related to health media campaigns.

^†^We reran the main analyses controlling for baseline adherence and COVID-19 cases per million on the day of data collection in that country, finding the same pattern of results as confirmatory analyses. We report these analyses in *SI Appendix*, Table S4 and focus the text on confirmatory analyses without these exploratory covariates.

^‡^We conducted exploratory analyses prior to peer review that focused on countries with available data in April 2020. These analyses found the same pattern of results and are described in prior preprint versions: https://psyarxiv.com/n3dyf/.

^§^This reflects the number of laboratories with which the PSACR has ethics documentation for data collection; it is possible that a small number of laboratories may have collected data for another PSACR study (001 or 002) but not ours (003).

^¶^All PSA and SDT researchers made at least two contributions to the study (data collection, study design, translation efforts, analysis, reviewing code, study administration, or writing) and approved the manuscript’s submission in order to be included as coauthors. Each author’s contribution is in the *SI Appendix*.
