# Supplementary material for: A global experiment on motivating social distancing during the COVID-19 pandemic
Source: Proc Natl Acad Sci U S A. 2022 May 27;119(22):e2111091119. doi: 10.1073/pnas.2111091119 (PMC9295806; doi:10.1073/pnas.2111091119)
Supplement: Supplementary File [file pnas.2111091119.sapp.pdf]

Supplemental Information Appendix

This document contains a) the results for baseline adherence and manipulation check analyses, b) more information about our design and procedure, translation process, and data exclusions and analytical strategy, c) a table of sample sizes for each country, d) analyses with two exploratory covariates, and e) a list of all the co-authors that are part of the Psychological Science Accelerator Rapid-Response COVID-19 Project along with all authors' contributions.

Results

Quality Checks

First, we examined whether the three conditions differed on a baseline measure of how much participants were already adhering to social distancing recommendations. Because differences across conditions were negligible (r_p = .014 and .008, see *Table S2* below) and lower than our preregistered threshold, baseline adherence was not included as a covariate in confirmatory models. Second, as this study was bundled with one other study in the Psychological Science Accelerator Covid-19 Rapid (PSACR) project, we examined potential carryover effects based on the order of presentation (our study was presented first or was the only study participants took part in for n = 17,729, coded 0; or, it was presented after the bundled PSACR001 study, n = 7,989, coded 1). We tested the main effect of study order and the interaction with condition to examine whether the study order effect differentially impacted the effect of condition on the manipulation check (i.e., perceptions of social distancing recommendations as controlling). Effects surpassed our preregistered threshold, r_p ranging from .035–.043 (see *Table S2*), suggesting an effect of order. We tested models excluding participants who were exposed to the other PSACR study -— PSACR001 — first and found the same pattern of results. We present these subsample results in *Table S3* below and present the results from the full sample in the manuscript main text. This switch to

present full sample models in the main text represents a deviation from the preregistration; we did this to retain participants from as many countries as possible since we observed the same pattern of results.

Manipulation Check

Following this, we examined condition effects on the manipulation check, how controlling participants perceived the social distancing recommendations to be. The controlling message was perceived as more pressuring than both the autonomy-supportive message, r_p = .163, 95% CI [.176, .152], and general social distancing recommendations assessed in the no-message condition, r_p = .096, 95% CI [.108, .083].

Methods

Design and Procedure

This project deviated from the described methods of the PSACR project in that additional data collection labs were recruited through a network of self-determination theory (SDT) researchers. SDT labs were given a Qualtrics link for their lab's survey[1] whereas Psychological Science Accelerator (PSA) labs' data collection was centralized across labs with one survey using the *formr* (1) framework. Participants recruited by SDT labs only completed this study, and participants recruited by PSA labs completed this and a second, separate, PSACR experiment (coded as PSACR001)[2]. For those who participated in both studies, order was recorded to examine for possible carry-over effects.

Translations

[1] Two SDT labs (in Iran and Thailand) were not able to use Qualtrics for data collection because of restrictions and used Google Forms to collect data. Because of this difference in survey format, there were irregularities for the six-month behavioral intentions items. For analyses with this six-month behavioral intentions variable, the Thai lab was excluded (*n* = 240) because they measured items on a 1-7 scale (versus 1-24 weeks), and 10 individuals in the Iranian lab were excluded because they indicated a number higher than 24 [weeks] in the open-ended textbox.

[2] There was a period of approximately one month at the start of data collection (April, 2020) when this study (coded as PSACR003) was the only PSACR study collecting data and thus all participants recruited through PSA labs in April took part only in the present study.

All study materials were translated into the modal local language of participants recruited by each lab. The PSA identified translators for every language represented in the pool of collaborators, except for Thai, which was translated by a SDT lab. In all countries where participants' modal native language was not English, study materials were forward- and backward-translated by two or more translators (2). Once the translators agreed on a translation, the translated materials were sent to labs for any further cultural adjustment to their local context (e.g., differences between European and Brazilian Portuguese). In total, the survey was translated into 42 languages and regional dialects.

Measures

Baseline Adherence

Before random assignment to condition, participants answered questions about how much they were "currently following the recommendation to stay at home as much as possible" and how much they were following recommendations to avoid three specific situations: "gatherings with friends", "crowded areas", and "non-essential shopping trips". Participants rated their responses on a 7-point scale (1 = *not at all*, 7 = *completely*). The scale showed acceptable reliability for all items combined (ω = .87). Higher scores indicate greater adherence to social distancing guidance at baseline.

Perceived Control (Manipulation Check)

At the end of the study, participants responded to three items that assessed how autonomy-supportive versus controlling participants perceived the social distancing recommendations to be. These items were adapted from the Health Care Climate Questionnaire (3, 4). Participants in the autonomy-supportive and controlling message conditions responded to the stem "I feel that social distancing recommendations [in this article]" paired with the items: "...provided people some choices and options", "...try to pressure people", and "...aren't very sensitive to people's needs". Participants in the no-

message condition responded to the same stem with the words “in this article” excluded. Participants rated their responses on a 7-point scale (1 = *strongly disagree*, 7 = *strongly agree*). These three items showed less than acceptable reliability (ω = .62), so, following the preregistered procedure, we retained the two items (the second and third items above) that showed corrected item-total correlations above .30. Higher scores indicated perceiving recommendations as more controlling (ω = .67).

Analytic Strategy

Data Exclusions

Several basic cleaning steps were applied to the raw data (see https://osf.io/uzqdr/): duplicate entries were removed, test sessions that occurred before the survey launched in each language were removed, the data were deidentified, and blank observations produced by the survey software were removed.

The preregistration describes a procedure for excluding data on the basis of completion time only. We deviated from the preregistration by adding three additional exclusion criteria that rendered the data unfit for our analyses. Our procedure was approved by ethics boards for participants over 18. We excluded data from participants who were less than 18 years old (n = 30). We also excluded data from participants who did not fill out at least 75% of the outcome measures ([motivation, feelings of defiance, behavioral intentions, and the manipulation check]; n = 1,442). Of participants who did not fill out at least 75% of the outcome measures, most filled out no outcome measures (n = 1,409). Finally, we excluded data from participants whose country could not be identified (n = 692). After these exclusions, there were no participants who were excluded for fast responding (less than two standard deviations of the mean time of completion within each condition). See *Figure 1* in the main text delineating the final sample.

Modelling Approach

Before fitting models, we ensured there was sufficient variance at both individual and country-levels. Intraclass correlation coefficients ranged from .03 -.15, indicating that while sufficient for mixed-effects models, at most only 15% of variance was explained by between-country differences. Most of the variance was between individuals within country.

When fitting maximal models, or models with random slopes, we excluded countries with sample sizes below 210 to help with model convergence. This yielded a sample of 23,557 individuals in 35 countries for analyses with random slopes. Comparisons of the Akaike information criterion — an estimator of prediction error that evaluates how well a model fits the data [3] — indicated that the maximal models fit the data better than the intercept-only models. Nonetheless, we did not observe large variance around the slopes of condition (see *Table 3* in the main text). Because of little variance around the slopes in some models, some models failed to converge. This indicates that effects of our manipulation did not vary widely across these 35 countries, even though countries might differ in their averages on the measured variables.

To test Hypothesis 1, whether those in the no-message and autonomy-supportive message conditions differed on outcomes as compared to those exposed to a controlling message, we created two dummy variables. The autonomy-supportive message and no-message conditions (entered simultaneously into models) were both coded 1, and the controlling message condition was the reference group, coded 0. For Hypothesis 2, we examined whether self-reported autonomous and controlled motivation, also entered simultaneously, predicted outcomes (defiance and short and long-term behavioural intentions) using mixed-effects models.

References

1. Arslan, R. C., M. P. Walther, C. S. Tata, formr: A study framework allowing for automated feedback generation and complex longitudinal experience-sampling studies using R. *Behavior Research Methods* **52**, 376–387 (2020).
2. Brislin, R. W. (1970). *Back-translation for cross-cultural research* (1971-06132-001; Issues 2-B) [ProQuest Information & Learning]. APA PsycInfo. https://ezproxy.gl.iit.edu/login?url=http://search.ebscohost.com/login.aspx?direct=true&db=psyh&AN=1971-06132-001&site=ehost-live
3. Kasser, V. G., R. M. Ryan, The Relation of Psychological Needs for Autonomy and Relatedness to Vitality, Well-Being, and Mortality in a Nursing Home1. *J Appl Social Pyschol* **29**, 935–954 (1999).
4. Williams, G. C., Cox, E. M., Kouides, R., Deci, E. L. Presenting the Facts About Smoking to Adolescents: Effects of an Autonomy-Supportive Style. *Arch Pediatr Adolesc Med* **153**, 959 (1999).
5. T. Hale, *et al.*, A global panel database of pandemic policies (Oxford COVID-19 government response tracker). *Nat. Hum. Behav.* **5**, 529–538 (2021).

Table S1

List of countries/regions (alphabetized by country/region) and their respective stringency measures implementation date and sample sizes collected through the PSA and SDT networks.

Country/region name	Stringency date	PSA (n = 18,710)	SDT (n = 7,008)	Total (n = 25,718)
Albania	14/03/2020	2		2
Algeria	22/03/2020	2		2
Argentina	22/03/2020	124		124
Armenia	N/A	17		17
Australia	23/03/2020	539	1128	1667
Austria	15/03/2020	527		527
Azerbaijan	22/03/2020	1		1
Bahrain	27/03/2020	1		1
Bangladesh	25/03/2020	21		21
Belarus	07/04/2020	4		4
Belgium	19/03/2020	104	1010	1114
Bosnia and Herzegovina	19/03/2020	22		22
Brazil	20/03/2020	105		105
British Virgin Islands	N/A	1		1
Brunei	23/03/2020	1		1
Bulgaria	20/03/2020	83		83
Canada	22/03/2020	258	253	511
Cape Verde	31/03/2020	1		1
Chile	24/03/2020	278	401	679
China	31/01/2020	468		468
Colombia	24/03/2020	109		109
Costa Rica	20/03/2020	191		191
Croatia	22/03/2020	945		945
Cyprus	23/03/2020	5		5
Czechia	22/03/2020	213		213
Denmark	13/03/2020	3		3
Ecuador	16/03/2020	18		18
Egypt	24/03/2020	612		612
Finland	17/03/2020	147	236	383
France	16/03/2020	653		653
Germany	21/03/2020	294	235	529
Greece	22/03/2020	70	55	125
Guadeloupe	N/A	1		1
Guam	N/A	1		1
Hong Kong	28/01/2020	28		28
Hungary	15/03/2020	97		97

Iceland	19/03/2020	1		1
India	21/03/2020	35		35
Iran	04/03/2020	67	184	251
Ireland	27/03/2020	55		55
Israel	16/03/2020	118		118
Italy	04/03/2020	124	552	676
Japan	01/03/2020	1064		1064
Jordan	17/03/2020		450	450
Kazakhstan	28/03/2020	3		3
Kenya	23/03/2020	671		671
Kosovo	23/03/2020	1		1
Lebanon	17/03/2020	1		1
Lithuania	15/03/2020	1		1
Luxembourg	16/03/2020	1		1
Malaysia	17/03/2020	52		52
Mexico	29/03/2020	387		387
Moldova	22/03/2020	7		7
Montenegro	N/A	3		3
Morocco	24/03/2020	63		63
Mozambique	31/03/2020	1		1
Netherlands	22/03/2020	114	290	404
New Zealand	25/03/2020	140		140
Nigeria	28/03/2020	605		605
Macedonia	N/A	89		89
Norway	23/03/2020	314		314
Pakistan	25/03/2020	275		275
Peru	17/03/2020		413	413
Philippines	21/03/2020	272		272
Poland	14/03/2020	1623		1623
Portugal	18/03/2020	190		190
Qatar	21/03/2020	1		1
Romania	20/03/2020	371		371
Russia	16/03/2020	387		387
Saudi Arabia	20/03/2020	3		3
Serbia	20/03/2020	89		89
Singapore	09/04/2020	72		72
Slovakia	15/03/2020	128	39	167
Slovenia	19/03/2020	35		35
South Africa	25/03/2020	491		491

South Korea	03/02/2020	285		285
Spain	16/03/2020	5		5
Sweden	24/03/2020	354		354
Switzerland	16/03/2020	312	166	478
Thailand	17/03/2020	8	240	248
Trinidad and Tobago	28/03/2020	1		1
Taiwan	N/A	63		63
Turkey	27/03/2020	237		237
Ukraine	17/03/2020	2		2
United Arab Emirates	26/03/2020	12		12
United Kingdom	23/03/2020	737	195	932
United States	20/03/2020	2892	1161	4053
Vietnam	31/03/2020	1		1
Yemen	29/04/2020	1		1

Note. PSA is the Psychological Science Accelerator network. SDT is the self-determination theory network. Stringency date refers to the date that each country implemented measures such as lockdowns and school closures according to the Oxford COVID-19 Government Response Tracker[5].

Table S2

Random intercept models testing condition equivalence on a baseline measure of social distancing adherence, carryover effects, and the effect of condition on the manipulation check

							95% CI around r_p			Variance of
**Outcome**	**term**	***B***	***SE***	***t***	***df***	r_p	**Lower**	**Upper**	***p***	**random effects**
Baseline adherence	Controlling (intercept)	5.11	.08	64.20	72.78	.014	.004	.027	<.001	.392
	vs. No message	.06	.02	2.40	25654.61	.014	.002	.026	.017	
	vs. Autonomy supportive	.03	.02	1.41	25651.47	.008	.000	.020	.167	
Carryover effect on manipulation check (perceived control)	Controlling (intercept)	4.21	.05	79.41	81.59	.170	.158	.182	<.001	.128
	vs. No message	-.50	.03	-15.98	24755.96	.099	.086	.111	<.001	
	vs. Autonomy supportive	-.82	.03	-25.92	24751.07	.159	.147	.171	<.001	
	Carryover (study order)	-.24	.04	-5.84	24804.00	.037	.025	.050	<.001	
	vs. No message x carryover	.31	.06	5.56	24758.02	.035	.022	.047	<.001	
	vs. Autonomy supportive x carryover	.39	.06	6.99	24758.26	.043	.031	.056	<.001	
Manipulation check (perceived control)	Controlling (intercept)	4.14	.05	80.76	70.86	.163	.152	.176	<.001	.128
	vs. No message	-.40	.03	-15.48	24760.64	.096	.083	.108	<.001	
	vs. Autonomy supportive	-.69	.03	-26.57	24752.80	.163	.151	.175	<.001	

Note. SE, standard error; *df,* degrees of freedom; *B*s are unstandardized coefficients; r_p is the partial standardized effect size for each coefficient; *CI,* Confidence Interval; *1w*, 1 week; *6m*, 6 months; *Excluding erroneous data;
N = 25,718; Controlling: *n* = 8,368; No message: *n* = 8,790; Autonomy supportive: *n* = 8,560; The controlling message was the reference group; We report three decimal places for r_p and its CI since our interval null is r_p = -.025 to .025, and two decimals for all other values.

Table S3

Random intercept-only models testing the effect of experimental conditions (Hypothesis 1) and autonomous and controlled motivation (Hypothesis 2), excluding participants who were presented with a different PSACR experiment first

							95% CI around r_p			Variance of
**Outcome**	**term**	***B***	***SE***	***t***	***df***	r_p	**Lower**	**Upper**	***p***	**random effects**
Baseline adherence	Controlling (intercept)	5.04	.08	60.38	73.23	.024	.011	.039	< .001	.402
	vs. No message	.09	.03	3.10	17674.87	.022	.007	.036	.002	
	vs. Autonomy supportive	.08	.03	2.80	17673.30	.019	.005	.034	.005	
Manipulation check (perceived control)	Controlling (intercept)	4.22	.06	76.36	71.17	.193	.179	.207	<.001	.134
	vs. No message	-.50	.03	-16.08	16979.25	-.120	-.134	-.105	<.001	
	vs. Autonomy supportive	-.82	.03	-26.05	16975.03	-.192	-.206	-.177	<.001	
Autonomous motivation Hypothesis 1	Controlling (intercept)	5.99	.06	103.45	72.64	.061	.047	.076	<.001	.191
	vs. No message	-.06	.02	-2.99	17668.28	-.021	-.036	-.006	.003	
	vs. Autonomy supportive	.11	.02	5.50	17666.59	.038	.024	.053	<.001	
Controlled motivation Hypothesis 1	Controlling (intercept)	4.57	.06	75.58	73.60	.105	.091	.120	<.001	.193
	vs. No message	-.36	.03	-14.20	17664.94	-.101	-.116	-.087	<.001	
	vs. Autonomy supportive	-.09	.03	-3.71	17662.85	-.027	-.041	-.012	<.001	
Defiance Hypothesis 1	Controlling (intercept)	2.80	.05	53.69	70.95	.086	.072	.101	<.001	.123
	vs. No message	-.02	.03	-.68	17482.80	-.005	-.020	-.000	.499	
	vs. Autonomy supportive	-.30	.03	-10.40	17480.46	-.077	-.091	-.062	<.001	
Defiance Hypothesis 2	Intercept	6.12	.07	81.66	390.19	.511	.501	.521	<.001	.113
	Autonomous motivation	-.73	.01	-75.90	17412.98	-.509	-.519	-.499	<.001	
	Controlled motivation	.22	.01	28.15	17487.76	.210	.196	.224	<.001	
Intention to avoid 1week Hypothesis 1	Controlling (intercept)	5.37	.07	75.20	74.92	.027	.014	.043	<.001	.281
	vs. No message	.10	.03	3.60	17344.40	.026	.011	.041	<.001	
	vs. Autonomy supportive	.08	.03	2.97	17341.69	.021	.006	.036	.003	
Intention to avoid 1week Hypothesis 2	Intercept	2.23	.08	27.78	290.95	.411	.399	.423	<.001	.171
	Autonomous motivation	.54	.01	56.56	17356.25	.399	.387	.411	<.001	
	Controlled motivation	.00	.01	-.63	17370.00	.005	.000	.020	.526	
Intention to avoid 6 months Hypothesis 1*	Controlling (intercept)	16.93	.28	59.97	72.69	.003	.002	.022	<.001	4.12
	vs. No message	-.05	.12	-.39	16805.33	-.003	-.018	-.000	.698	
	vs. Autonomy supportive	-.04	.12	-.37	16801.93	-.003	-.018	-.000	.710	
Intention to avoid 6 months Hypothesis 2*	Intercept	2.62	.33	7.97	365.16	.454	.443	.466	<.001	2.28
	Autonomous motivation	2.66	.04	64.23	16762.71	.453	.442	.464	<.001	
	Controlled motivation	-.39	.03	-11.80	16817.81	-.091	-.106	-.076	< .001	

Note. SE, standard error; *df,* degrees of freedom; *B*s are unstandardized coefficients; r_p is the partial standardized effect size for each coefficient; *CI,* Confidence Interval; *1w*, 1 week; *6m*, 6 months; *Excluding erroneous data; *N = 17,729;* Controlling: *n* = 5,733; No message: *n* = 6,099; Autonomy supportive: *n* = 5,897; The controlling message was the reference group; We report three decimal places for r_p and its CI since our interval null is r_p = -.025 to .025, and two decimals for all other values.

Table S4

Random intercept-only models testing effects of experimental conditions (Hypothesis 1) and autonomous and controlled motivation (Hypothesis 2) on outcomes with two exploratory covariates

Outcome	term	*B*	*SE*	*t*	*df*	r_p	95% CI around r_p Lower	Upper	*p*	Variance of random effects
Manipulation check (Perceived control)	Controlling (intercept)	4.83	.06	76.26	177.05	.209	.197	.221	<.001	.117
	vs. No message	-.40	.03	-15.44	24325.07	-.097	-.109	-.084	<.001	
	vs. Autonomy-supportive	-.70	.03	-26.87	24317.92	-.166	-.178	-.154	<.001	
	Baseline adherence	-.14	.01	-19.29	23984.63	-.127	-.139	-.115	<.001	
	Total cases per million	.00	.00	1.22	6082.97	.012	.001	.024	.224	
Autonomous motivation Hypothesis 1	Controlling (intercept)	4.66	.05	89.67	125.04	.364	.354	.374	<.001	.122
	vs. No message	-.05	.02	-3.21	25030.39	-.019	-.032	-.007	.001	
	vs. Autonomy-supportive	.09	.02	5.70	25028.01	.034	.022	.047	<.001	
	Baseline adherence	.26	.00	60.82	25064.62	.360	.350	.371	<.001	
	Total cases per million	.00	.00	1.58	17270.05	.015	.003	.027	.113	
Controlled motivation Hypothesis 1	Controlling (intercept)	4.16	.07	62.95	130.63	.134	.122	.146	<.001	.192
	vs. No message	-.35	.02	-16.62	25023.06	-.100	-.112	-.087	<.001	
	vs. Autonomy-supportive	-.10	.02	-4.73	25020.56	-.028	-.041	-.016	<.001	
	Baseline adherence	.07	.01	12.96	25049.62	.082	.070	.095	<.001	
	Total cases per million	.00	.00	2.79	16590.94	.026	.014	.039	.005	
Defiance Hypothesis 1	Controlling (intercept)	3.88	.06	69.86	199.58	.227	.216	.239	<.001	.081
	vs. No message	.00	.02	.07	24905.93	.000	.000	.014	.942	
	vs. Autonomy-supportive	-.25	.02	-10.44	24899.51	.065	-.077	-.053	<.001	
	Baseline adherence	-.22	.01	-33.48	24347.35	.215	-.227	-.204	<.001	
	Total cases per million	.00	.00	.48	4874.02	.005	.000	.017	.630	
Defiance Hypothesis 2	Intercept	6.32	.07	93.47	369.06	.530	.522	.538	<.001	.101
	Autonomous motivation	-.74	.01	-86.43	24867.53	-.489	-.498	-.480	<.001	
	Controlled motivation	.23	.01	35.15	24882.63	.220	.208	.232	<.001	
	Baseline adherence	-.04	.01	-6.11	24844.96	-.039	-.052	-.027	<.001	
	Total cases per million	.00	.00	.76	9054.44	.007	.000	.020	.450	
Intention to avoid 1w Hypothesis 1	Controlling (intercept)	2.84	.05	56.76	165.92	.548	.540	.556	<.001	.085
	vs. No message	.03	.02	1.83	24810.66	.011	.001	.024	.068	
	vs. Autonomy-supportive	.01	.02	.61	24806.20	.004	.000	.016	.542	
	Baseline adherence	.51	.01	100.17	24686.01	.548	.540	.556	<.001	
	Total cases per million	.00	.00	-1.54	9609.35	.015	.003	.027	.124	
Intention to avoid 1w Hypothesis 2	Intercept	1.09	.06	19.36	461.19	.618	.611	.625	<.001	.062
	Autonomous motivation	.37	.01	50.67	24730.85	.314	.303	.325	<.001	
	Controlled motivation	.01	.01	.95	24755.76	.006	.000	.019	.341	
	Baseline adherence	.41	.01	79.59	24705.42	.459	.450	.468	<.001	
	Total cases per million	.00	.00	-2.10	7714.31	.020	.008	.033	.035	
Intention to avoid 6m Hypothesis 1*	Controlling (intercept)	10.33	.25	40.74	157.10	.353	.342	.364	<.001	2.240
	vs. No message	-.12	.09	-1.30	24427.45	-.008	-.021	-.000	.195	
	vs. Autonomy-supportive	-.26	.10	-2.71	24422.94	-.017	-.029	-.004	.007	
	Baseline adherence	1.44	.03	56.31	24329.53	.347	.336	.358	<.001	
	Total cases per million	.00	.00	-8.36	9780.53	.081	.068	.093	<.001	
Intention to avoid 6m Hypothesis 2*	Intercept	1.05	.28	3.74	385.92	.511	.503	.520	<.001	1.680
	Autonomous motivation	2.32	.04	64.46	24381.54	.389	.378	.399	<.001	
	Controlled motivation	-.42	.03	-15.46	24399.19	-.100	-.112	-.087	<.001	
	Baseline adherence	.87	.03	34.05	24359.35	.217	.205	.229	<.001	
	Total cases per million	.00	.00	-9.28	8324.73	.090	.077	.102	<.001	

Note. SE, standard error; *df,* degrees of freedom; *B*s are unstandardized coefficients; r_p is the partial standardized effect size for each coefficient; *CI,* Confidence Interval; *1w*, 1 week; *6m*, 6 months; *Excluding erroneous data*;*
N = *25,718;* Controlling: *n* = 8,368; No message: *n* = 8,790; Autonomy-supportive: *n* = 8,560; The controlling message was the reference group; We report three decimal places for r_p and its CI since our interval null is r_p = -.025 to .025, and two decimals for all other values.

Table S5
Authorship order within consortium (because of journal page limits, we were unable to list all authors individually and the order here reflects the intended author order).

PSACR Author	Institution and Affiliation	Email
Nicole Legate (co-first author)	Illinois Institute of Technology, Department of Psychology, Chicago, Illinois, United States	nlegate@iit.edu
Thuy-vy Ngyuen (co-first author)	Durham University, Department of Psychology, Durham, United Kingdom	thuy-vy.nguyen@durham.ac.uk
Netta Weinstein	University of Reading, Department of Psychology, Reading, United Kingdom	n.weinstein@reading.ac.uk
Arlen Moller	Illinois Institute of Technology, Department of Psychology, Chicago, Illinois, United States	amoller@iit.edu
Lisa Legault	Clarkson University, Department of Psychology, Potsdam, New York, United States	llegault@clarkson.edu
Zahir Vally	United Arab Emirates University, Al Ain, United Arab Emirates	zahir.vally@uaeu.ac.ae
	Wolfson College, University of Oxford, Oxford, United Kingdom	
Zuzanna Tajchman	University of Minnesota, Twin Cities, Department of Psychology, Minneapolis, United States	ztajchma@umn.edu
Andras N. Zsido	Institute of Psychology, University of Pécs, Pécs, Hungary	zsido.andras@pte.hu
Miha Zrimsek	University of Ljubljana, Department of Translation Studies, Faculty of Arts , Ljubljana, Slovenia	zrimsekm@gmail.com
Zhang Chen	Ghent University, Department of Experimental Psychology, Ghent, Belgium	zhang.chen@ugent.be
Ignazio Ziano	Grenoble Ecole de Management, Grenoble, France	ziano.ignazio@gmail.com
Zoi Gialitaki	Independent Researcher	z.gialitaki@gmail.com
Chris D Ceary	Indiana University of Pennsylvania, Indiana, United States	cceary@gmail.com
Yuna Jang	Independent Researcher	yunajang92@gmail.com
Yijun Lin	University of Florida, Department of Psychology, Gainesville, United States	yijun.lin@ufl.edu

Yoshihiko Kunisato	Senshu University, Department of Psychology, Kawasaki , Japan	ykunisato@psy.senshu-u.ac.jp
Yuki Yamada	Kyushu University, Faculty of Arts and Science, Fukuoka, Japan	yamadayuk@gmail.com
Qinyu Xiao	University of Hong Kong, Department of Psychology, Hong Kong SAR, China	xqy1020@connect.hku.hk
Xiaoming Jiang	Shanghai International Studies University, Institute of Linguistics, Shanghai, China	xiaoming.jiang@shisu.edu.cn
Xinkai Du	University of Amsterdam, Amsterdam, Netherlands	xinkai.du@student.uva.nl
Elvin Yao	Claremont Graduate University, Claremont, United States	xiaohui.yao@cgu.edu
William S. Ryan	University of Toronto, St George, Canada	ws.ryan@utoronto.ca
John Paul Wilson	Montclair State University, Montclair, United States	johnpaulw@gmail.com
Wilson Cyrus-Lai	INSEAD, Singapore	wilson-cyrus.lai@insead.edu
William Jimenez-Leal	Universidad de los Andes, Department of Psychology, Bogotá, Colombia	w.jimenezleal@uniandes.edu.co
Wilbert Law	The Education University of Hong Kong, Department of Psychology, Hong Kong, SAR China	wlaw@eduhk.hk
Wenceslao Unanue	Universidad Adolfo Ibáñez, School of Business, Santiago, Chile	wenceslao.unanue@uai.cl
W. Matthew Collins	Nova Southeastern University, Department of Psychology and Neuroscience, Fort Lauderdale, United States	wc292@nova.edu
Karley L Richard	Indiana University of Pennsylvania, Indiana, United States	vymbc@iup.edu
Marek Vranka	Charles University, Prague, Czechia	vranka.marek@gmail.com
Vladislav Ankushev	HSE University, Moscow, Russia	vladislavankushev@yandex.ru
Vidar Schei	NHH Norwegian School of Economics, Department of Strategy and Management, Bergen, Norway	vidar.schei@nhh.no
Chloe DePaola	Indiana University of Pennsylvania, Indiana, United States	vhvp@iup.edu
Veronika Lerche	Heidelberg University, Heidelberg, Germany	veronika.lerche@psychologie.uni-heidelberg.de

Vanja Kovic	University of Belgrade, Laboratory for Neurocognition and Applied Cognition, Faculty of Philosophy, Belgrade, Serbia	vanja.kovic@f.bg.ac.rs
Valerija Križanić	Josip Juraj Strossmayer University of Osijek, Department of Psychology, Faculty of Humanities and Social Sciences, Osijek, Croatia	vkrizanic@ffos.hr
Veselina Hristova Kadreva	New Bulgarian University, Department of Cognitive Science and Psychology, Sofia, Bulgaria	v.kadreva@gmail.com
Vera Cubela Adoric	University of Zadar, Department of Psychology, Zadar, Croatia	vcubela@unizd.hr
Ulrich S. Tran	University of Vienna, Department of Cognition, Emotion, and Methods in Psychology, Faculty of Psychology, Vienna, Austria	ulrich.tran@univie.ac.at
Siu Kit Yeung	The University of Hong Kong, Hong Kong, SAR China	u3517520@connect.hku.hk
Widad Hassan	University of East London, Department of Psychology, Dubai, United Arab Emirates	u1944146@uel.ac.uk
Ralph Houston	Independent Researcher	translate@rjfhouston.com
Michael A. Machin	University of Southern Queensland, Toowoomba, Australia	tony.machin@usq.edu.au
Tiago J. S. Lima	University of Brasília, Department of Social and Work Psychology, Brasilia, Brazil	tiago.lima@unb.br
Thomas Ostermann	Witten/Herdecke University, Department of Psychology and Psychotherapy, Witten, Germany	thomas.ostermann@uni-wh.de
Thomas Frizzo	Université de Lorraine, Université de Strasbourg, CNRS, BETA, Nancy, France	thomas.frizzo@gmail.com
Therese E Sverdrup	NHH Norwegian School of Economics, Department of Strategy and Management, Bergen, Norway	therese.sverdrup@nhh.no
Thea House	Macquarie University, Sydney, Australia<br>University of Bristol, Bristol, United Kingdom	thea.house@students.mq.edu.au
Tripat Gill	Wilfrid Laurier University, Lazaridis School of Business and Economics, Waterloo, Canada	tgill@wlu.ca

Maksim Fedotov	Russian Academy of Sciences, Institute for Linguistic Studies, St. Petersburg, Russia	tequila.lime@gmail.com
Tamar Paltrow	Independent Researcher	tepaltrow@aol.com
Teodor Jernsäther	Stockholm University, Department of Psychology, Stockholm, Sweden	teodor.jernsather@psychology.su.se
Tasnim Rahman	University of Dhaka, Dhaka, Bangladesh	tasnimrahman621@gmail.com
Tanya Machin	University of Southern Queensland, Toowoomba, Australia	Tanya.Machin@usq.edu.au
Maria Koptjevskaja-Tamm	Stockholm University, Department of Linguistics, Stockholm, Sweden	tamm@ling.su.se
Thomas J. Hostler	Manchester Metropolitan University, Department of Psychology, Manchester, United Kingdom	t.hostler@mmu.ac.uk
Tatsunori Ishii	Japan Women's University, Faculty of Integrated Arts and Social Sciences, Tokyo, Japan.	t.ishii1108@gmail.com
Barnabas Szaszi	ELTE Eötvös Loránd University, Institute of Psychology, Budapest, Hungary	szaszi.barnabas@ppk.elte.hu
Sylwia Adamus	Jagiellonian University, Institute of Psychology, Krakow, Poland	sylwiadamus@gmail.com
Lilian Suter	ZHAW Zurich University of Applied Sciences, School of Applied Psychology, Winterthur, Switzerland	lilian.suter@zhaw.ch
Suparpit M. von Bormann	Suranaree University of Technology, Nakhon Ratchasima, Thailand	suparpit@gmail.com
Sumaiya Habib	University of Dhaka, Department of Clinical Psychology, Dhaka, Bangladesh	sumaiyahabib14@gmail.com
Anna Studzinska	Icam Toulouse, Humanities Department, Toulouse, France	studzinna@gmail.com
Dragana Stojanovska	PSA Psihesko, Skopje, North Macedonia	stojanovskadragana.ds@gmail.com
Steve M. J. Janssen	University of Nottingham Malaysia School of Psychology, Semenyih, Malaysia	steve.janssen@nottingham.edu.my
Stefan Stieger	Karl Landsteiner University of Health Sciences, Department of Psychology and Psychodynamics, Krems an der Donau, Austria	stefan.stieger@kl.ac.at

Stefan E. Schulenberg	University of Mississippi, Department of Psychology, Oxford, Mississippi, United States<br><br>University of Mississippi, Clinical-Disaster Research Center, Oxford, Mississippi, United States	sschulen@olemiss.edu
Srinivasan Tatachari	T A Pai Management Institute, Manipal Academy of Higher Education, Karnataka, India	srini.tata@gmail.com
Soufian Azouaghe	Mohammed V University in Rabat, Department of Psychology, Rabat, Morocco Université Grenoble Alpes, LIP/PC2S, Grenoble, France	s.azouaghe@um5r.ac.ma
Piotr Sorokowski	University of Wroclaw, Institute of Psychology, Wroclaw, Poland	sorokowskipiotr@yahoo.co.uk
Agnieszka Sorokowska	University of Wroclaw, Institute of Psychology, Wroclaw, Poland	Sorokowska@gmail.com
Xin Song	University of Minnesota, Twin Cities, Department of Psychology, Minneapolis, United States	songx953@umn.edu
Sofie Morbée	Department of Developmental, Personality and Social Psychology, Ghent University	Sofie.Morbee@UGent.be
Savannah Lewis	Ashland University, Ashland, United States	slewis5920@gmail.com
Sladjana Sinkolova	PSA Psihesko, Skopje, North Macedonia	sinkolova.s@gmail.com
Dmitry Grigoryev	National Research University Higher School of Economics, Moscow, Russia	dgrigoryev@hse.ru
Shira Meir Drexler	Department of Neurology, Mauritius Hospital Meerbusch, Meerbusch, Germany	shira.meir@gmail.com
Shimrit Daches	Bar-Ilan University, Department of Psychology, Ramat Gan, Israel	shimrit.daches@biu.ac.il
Shelby L. Levine	McGill University, Montreal Canada	shelby.levine@mail.mcgill.ca
Shawn N. Geniole	University of the Fraser Valley, Department of Psychology, Abbotsford, Canada	shawngeniole@gmail.com
Shahunur Akter	University of Dhaka, Dhaka, Bangladesh	Shahinoorakter27@gmail.com
Selena Vračar	University of Belgrade, Department of Psychology, Belgrade, Serbia	selenavracar1410@gmail.com

Sébastien Massoni	Université de Lorraine, Université de Strasbourg, CNRS, BETA, Nancy, France	sebastien.massoni@gmail.com
Sebastiano Costa	Università degli Studi della Campania Luigi Vanvitelli, Caserta, Italy	sebastiano.costa@unicampania.it
Saša Zorjan	University of Maribor, Department of Psychology, Maribor, Slovenia	sasa.zorjan1@um.si
Eylul Sarioguz	Sapienza University of Rome, Doctoral School of Psychological Sciences, Department of Developmental Psychology and Socialization Processes, Rome, Italy	sarioguzeylul@gmail.com
Sara Morales Izquierdo	University of Warwick, Coventry United Kingdom	sara.morales-izquierdo@warwick.ac.uk
Sarah Suzette Tshonda	Independent Researcher	sarahsuzette91@gmail.com
Sara G Alves	University of Porto, Center for Psychology at University of Porto, Porto, Portugal	up201304933@edu.fpce.up.pt
Sara Pöntinen	Åbo Akademi University, Faculty of Arts, Psychology and Theology, Turku, Finland	sara.pontinen@gmail.com
Sara Álvarez Solas	Universidad Regional Amazónica Ikiam, Grupo de investigación en Biogeografía y Ecología Espacial (BioGeoE2), Tena, Ecuador	sara.alvarez.solas@gmail.com
Santiago Ordoñez-Riaño	University of Guadalajara, Guadalajara, Colombia	santiagoordz@gmail.com
Sanja Batić Očovaj	Union University, Faculty of Legal and Business Studies Dr Lazar Vrkatic, Department of Psychology, Novi Sad, Serbia	sanja.batic@gmail.com
Sandersan Onie	Black Dog Institute, Sydney, Australia<br>University of New South Wales, School of Psychology, Sydney, Australia<br>Emotional Health for All Foundation, Jakarta, Indonesia	sandy.onie@gmail.com
Samuel Lins	University of Porto, Center for Psychology at University of Porto, Porto, Portugal	samuellins@fpce.up.pt
Theresa Biberauer	University of Cambridge, Cambridge, United Kingdom<br>Stellenbosch University, Stellenbosch, South Africa	samtb23@gmail.com

	University of the Western Cape, Cape Town, South Africa	
Sami Çoksan	Erzurum Technical University, Department of Psychology, Erzurum, Turkey	sami.coksan@erzurum.edu.tr
Sakda Khumkom	Suranaree University of Technology, Nakhon Ratchasima, Thailand	sakdakh@sut.ac.th
Asli Sacakli	Independent Researcher	sacakliasli@gmail.com
Susana Ruiz-Fernández	FOM University of Applied Sciences, Essen, Germany	s.ruiz-fernandez@iwm-tuebingen.de
Sandra J. Geiger	University of Amsterdam, Department of Psychology, Faculty of Social and Behavioural Sciences, Amsterdam, Netherlands	sandra.geiger@univie.ac.at
Saeideh FatahModares	Urmia University, Department of Sport Management, Faculty of Physical Education and Sport Science, Urmia, Iran	s.fmodares@yahoo.com
Radoslaw B. Walczak	University of Opole, Institute of Psychology, Opole, Poland	rwalczak@uni.opole.pl
Ruben Betlehem	Josip Juraj Strossmayer University of Osijek, Faculty of Humanities and Social Sciences, Department of Psychology, Osijek, Croatia	rbetlehem@ffos.hr
Roosevelt Vilar	Universidade Cruzeiro do Sul, São Paulo, Brazil	roosevelt.vilar@gmail.com
Rodrigo A. Cárcamo	University of Magallanes, Department of Psychology, Punta Arenas, Chile	rodrigo.carcamo@umag.cl
Robert M Ross	Macquarie University, Department of Psychology, Sydney, Australia	robross46@gmail.com
Randy McCarthy	Northern Illinois University Department of Psychology, DeKalb, United States	rmccarthy3@niu.edu
Tonia Ballantyne	Indiana University of Pennsylvania, Indiana, United States	rjxx@iup.edu
Erin C. Westgate	University of Florida, Department of Psychology, Gainesville, United States	erinwestgate@ufl.edu
Richard M. Ryan	Australian Catholic University, Institute for Positive Psychology and Education, Sydney, Australia	richard.ryan@acu.edu.au
Rafael Gargurevich	Pontifical Catholic University of Peru, Lima, Peru	rgargurevich@pucp.pe

Reza Afhami	Tarbiat Modares University, Department of Art Studies, Tehran, Iran	Afhami@modares.ac.ir
Dongning Ren	Tilburg University, Department of Social Psychology, Tilburg, Netherlands	d.ren@uvt.nl
Renan P. Monteiro	Federal University of Mato Grosso, Department of Psychology, Cuiabá, Brazil	renanpmonteiro@gmail.com
Ulf-Dietrich Reips	University of Konstanz, Department of Psychology, Konstanz, Switzerland	reips@uni-konstanz.de
Niv Reggev	Ben Gurion University, Department of Psychology and Zlotowski Center for Neuroscience, Beersheba, Israel	reggevn@bgu.ac.il
Robert J Calin-Jageman	Dominican University, Department of Psychology, River Forest, United States	rcalinjageman@dom.edu
Razieh Pourafshari	University of Tehran, Department of Psychology, Faculty of Psychology and Education, Tehran, Iran	razieh.pourafshari@gmail.com
Raquel Oliveira	Iscte-Instituto Universitário de Lisboa, CIS-IUL, Lisbon, Portugal<br>Intelligent Agents and Synthetic Characters Group (GAIPS), INESC-ID, Lisbon, Portugal	rsaoa@iscte-iul.pt
Mina Nedelcheva-Datsova	Sofia University St. Kliment Ohridski, Department of General, Experimental, Developmental, and Health Psychology, Sofia, Bulgaria	raiskopruskalo@gmail.com
Rima-Maria Rahal	Max Planck Institute for Research on Collective Goods, Bonn, Germany<br>Tilburg University, Tilburg, Netherlands	rahal@coll.mpg.de
Rafael R Ribeiro	Iscte - Instituto Universitário de Lisboa, CIS-IUL, Lisbon, Portugal	rafael_ribeiro@iscte-iul.pt
Theda Radtke	University of Wuppertal, Department of Psychology, Wuppertal, Germany	Radtke@uni-wuppertal.de
Rachel Searston	The University of Adelaide, Adelaide, Australia	rachel.searston@adelaide.edu.au
Rachadaporn Jai-ai	Suranaree University of Technology, Nakhon Ratchasima, Thailand	rachadaporn@sut.ac.th
Redeate Habte	Jacobs University Bremen, Bremen, Germany	redhabte@gmail.com

Przemysław Zdybek	University of Opole, Institute of Psychology, Opole, Poland	pzdybek@uni.opole.pl
Sau-Chin Chen	Tzu-Chi University, Department of Human Development and Psychology, Hualien, Taiwan	pmsp96@gmail.com
Piyaorn Wajanatinapart	Suranaree University of Technology, Nakhon Ratchasima, Thailand	piyaorn@g.sut.ac.th
Princess Lovella G. Maturan	University of the Philippines Diliman, Department of Psychology, Quezon City, Philippines	pgmaturan@up.edu.ph
Jennifer T Perillo	Indiana University of Pennsylvania, Department of Psychology, Indiana, United States	jperillo@iup.edu
Peder Mortvedt Isager	Eindhoven University of Technology, Department of Industrial Engineering and Innovation Sciences, Eindhoven, Netherlands	pederisager@gmail.com
Pavol Kačmár	Pavol Jozef Šafárik University in Košice, Department of Psychology, Faculty of Arts, Košice, Slovakia	pavol.kacmar@upjs.sk
Paulo Manuel Macapagal	Arellano University, School of Psychology, Manila, Philippines	paulo.macapagal@arellano.edu.ph
Michael R. Maniaci	Department of Psychology, Florida Atlantic University	mmaniaci@fau.edu
Paulina Szwed	Jagiellonian University, Krakow, Poland	paulina.szwed@uj.edu.pl
Paul H. P. Hanel	University of Essex, Essex, United Kingdom	p.hanel@essex.ac.uk
Paul A G Forbes	University of Vienna, Social, Cognitive and Affective Neuroscience Unit, Department of Cognition, Emotion, and Methods in Psychology, Faculty of Psychology, Vienna, Austria	paul.forbes@univie.ac.at
Patricia Arriaga	Iscte - Instituto Universitário de Lisboa, CIS-IUL, Lisbon, Portugal	patricia.arriaga@iscte-iul.pt
Bastien Paris	Université Grenoble Alpes, Grenoble, France	paris.bastien@hotmail.com
Neha Parashar	Sampurna Montfort College, Bangalore, India	parashar3@gmail.com
Konstantinos Papachristopoulos	Concordia University, Montreal, Canada<br>Athens University of Economics and Business, Athens, Greece	papachristopouloskostas@gmail.com

Pablo Sebastián Correa	Universidad Nacional de Córdoba (UNC), Facultad de Psicología; Instituto de Investigaciones Psicológicas (IIPsi) - Consejo Nacional de Investigaciones Científicas y Técnicas (CONICET) - UNC, Córdoba, Argentina	pablocorrea@unc.edu.ar
Ondřej Kácha	University of Cambridge, Department of Psychology, Cambridge, United Kingdom	oek22@cantab.ac.uk
Márcia Bernardo	Universidade do Porto, Faculdade de Psicologia e Ciências da Educação,, Porto, Portugal	oliviabernardo95@gmail.com
Olatz Campos	University of Deusto, Bilbao, Spain	olatzcampos4@gmail.com
Olalla Niño Bravo	Independent Researcher	olallanino3@gmail.com
Oscar J Galindo-Caballero	Universidad de los Andes, Department of Psychology, Bogotá, Colombia<br>Universidad Manuela Beltran, Faculty of Education, Human and Social Sciences, Bogotá, Colombia.	oj.galindo10@uniandes.edu.co
Chisom Esther Ogbonnaya	Alex Ekwueme Federal University, Ndufu-Alike, Nigeria	ogbonnaya.chisom@yahoo.com
Olga Bialobrzeska	SWPS University of Social Sciences and Humanities, Warsaw, Poland	obialobrzeska@swps.edu.pl
Natalia Kiselnikova	Psychological Institute of Russian Academy of Education, Moscow, Russia	nv_psy@mail.ru
Nicolle Simonovic	Kent State University, Department of Psychological Sciences, Kent, United States	nsimonov@kent.edu
Noga Cohen	University of Haifa, Department of Special Education and The Edmond J. Safra Brain Research Center for the Study of Learning Disabilities, Haifa, Israel	noga.cohen@edu.haifa.ac.il
Nora L. Nock	Case Western Reserve University, Department of Population and Quantitative Health Sciences, Cleveland, Ohio, United States	nln@case.edu
Alejandrina Hernandez	Universidad Nacional Autonoma de Mexico, Mexico City, Mexico	nina.hp@hotmail.com
Cecilie Thogersen-Ntoumani	University of Southern Denmark, Department of Sports Sciences and Clinical Biomechanics, Odense, Denmark	cthogersen@health.sdu.dk

Nikos Ntoumanis	University of Southern Denmark, Department of Sports Sciences and Clinical Biomechanics, Odense, Denmark	nntoumanis@health.sdu.dk
Niklas Johannes	University of Oxford, Oxford Internet Institute, Oxford, United Kingdom	niklas.johannes@oii.ox.ac.uk
Nihan Albayrak-Aydemir	London School of Economics and Political Science, London, United Kingdom & Open University, Milton Keynes, United Kingdom	n.albayrak1@lse.ac.uk
Nicolas Say	Prague University of Economics and Business, Prague, Czechia	sayn00@vse.cz
Andreas B. Neubauer	DIPF | Leibniz Institute for Research and Information in Education, Frankfurt am Main, Germany	neubauer.andreas@dipf.de
Neil I. Martin	University of Southern Queensland, Toowoomba, Australia	neil.martin@usq.edu.au
Neil Levy	Macquarie University, Department of Philosophy, Sydney, Australia	neil.levy@mq.edu.au
Nathan Torunsky	University of Minnesota,Twin Cities, Department of Psychology, Minneapolis, United States	torun005@umn.edu
Natasha van Antwerpen	University of Adelaide, Adelaide, Australia	vananata10@gmail.com
Natalia Van Doren	The Pennsylvania State University, Department of Psychology, State College, United States	nataliavandoren@psu.edu
Naoyuki Sunami	University of Delaware, Newark, United States	nsunami@udel.edu
Nikolay R. Rachev	Sofia University St. Kliment Ohridski, Department of General, Experimental, Developmental, and Health Psychology, Sofia, Bulgaria	nrrachev@phls.uni-sofia.bg
Nadyanna M Majeed	Singapore Management University, School of Social Sciences, Singapore	nadyannam.2020@msps.smu.edu.sg
Nadya-Daniela Schmidt	University of Hildesheim, Institute of Psychology, Hildesheim, Germany	schmidtn@uni-hildesheim.de
Khaoula Nadif	Independent Researcher	nadifkhaoula@gmail.com
Nadia S Corral-Frías	Universidad de Sonora, Sonora, Mexico	nadia.corral@unison.mx
Nihal Ouherrou	Paul Valery Montpellier 3 University, Lhumain Laboratory, Montpellier, France	nihal.ouherrou@umontpellier.fr

Nida Abbas	Jacobs University Bremen, Bremen, Germany	adinxxabbas@gmail.com
Myrto Pantazi	University of Oxford, Oxford Internet Institute, Oxford, United Kingdom	myrto.pantazi@oii.ox.ac.uk
Marc Y Lucas	Universidad de Sonora, Department of Psychology, Sonora, Mexico	mylucas@email.arizona.edu
Martin R. Vasilev	Bournemouth University, Department of Psychology, Poole, United Kingdom	mvasilev@bournemouth.ac.uk
María Victoria Ortiz	Universidad Nacional de Córdoba (UNC), Facultad de Psicología; Instituto de Investigaciones Psicológicas (IIPsi) - Consejo Nacional de Investigaciones Científicas y Técnicas (CONICET) - UNC, Córdoba, Argentina	mv.ortiz@unc.edu.ar
Muhammad Mussaffa Butt	Government College University, Lahore, Pakistan	mussaffa@gmail.com
Murathan Kurfalı	Stockholm University, Linguistics Department, Stockholm, Sweden	murathan.kurfali@ling.su.se
Muhib Kabir	Bangladesh Clinical Psychology Society, Dhaka, Bangladesh	Muhib_cu@yahoo.com
Rafał Muda	Maria Curie-Sklodowska University, Faculty of Economics, Lublin, Poland	muda.research@gmail.com
María del Carmen MC Tejada Rivera	University of Desarrollo, Faculty of Psychology, Santiago, Chile	mtejadar@udd.cl
Miroslav Sirota	University of Essex, Essex, United Kingdom	msirota@essex.ac.uk
Martin Seehuus	Middlebury College, Department of Psychology, Middlebury, United States<br>University of Vermont, Vermont Psychological Services, Burlington, United States	mseehuus@middlebury.edu
Michal Parzuchowski	SWPS University of Social Sciences and Humanities in Sopot, Center for Research on Cognition and Behavior , Sopot, Poland	mparzuchowski@swps.edu.pl
Mónica Toro	University of Desarrollo, Faculty of Psychology, Santiago, Chile	motorov@udd.cl
Monika Hricova	Pavol Jozef Šafárik University in Košice, Department of Psychology, Faculty of Arts, Košice, Slovakia	monika.hricova@upjs.sk

Mónica Alarcón Maldonado	Independent Researcher	
Panagiotis Rentzelas	Department of Psychology and Human Development, IOE UCL's Faculty of Education and Society, University College London, London, United Kingdom	p.rentzelas@ucl.ac.uk
Maarten Vansteenkiste	Department of Developmental, Personality and Social Psychology, Ghent University	Maarten.Vansteenkiste@ugent.be
Molly A. Metz	University of Toronto, Toronto, Canada	molly.metz@utoronto.ca
Magdalena Marszalek	SWPS University of Social Sciences and Humanities, Warsaw, Poland	mmarszalek4@st.swps.edu.pl
Maria Karekla	University of Cyprus, Nicosia, Cyprus	mkarekla@ucy.ac.cy
Giovanna Mioni	University of Padova, Department of General Psychology, Padua, Italy	giovanna.mioni@unipd.it
Minke Jasmijn Bosma	University of Amsterdam, Department of Psychology, Amsterdam, Netherlands	minke.bosma@student.uva.nl
Minja Westerlund	Åbo Akademi University, Faculty of Arts, Psychology and Theology, Turku, Finland	minja.westerlund@abo.fi
Milica Vdovic	Singidunum University, Faculty of Media and Communications, Department of Psychology, Belgrade, Serbia	milica.vdovic@fmk.edu.rs
Michal Bialek	University of Wroclaw, Institute of Psychology, Wroclaw, Poland	michal.bialek3@uwr.edu.pl
Miguel A. Silan	University of the Philippines Diliman, Quezon City, Philippines	MiguelSilan@gmail.com
Michele Anne	University of Nottingham Malaysia, School of Psychology, Semenyih, Malaysia	michele.anne@dmu.ac.uk
Michal Misiak	University of Wroclaw, Institute of Psychology, Wroclaw, Poland<br>University of Oxford, School of Anthropology & Museum Ethnography, Oxford, United Kingdom	michal.misiak@uwr.edu.pl
Maria C. Gugliandolo	Università di Messina, Dipartimento DIMED, Messina, Italy	mariacristina.gugliandolo@unime.it

Maurice Grinberg	New Bulgarian University, Department of Cognitive Science and Psychology, Research Center for Cognitive Science, Sofia, Bulgaria	mgrinberg@nbu.bg
Mariagrazia Capizzi	Université Paul Valéry Montpellier 3, Montpellier, France	mgcapizzi@hotmail.com
Mauricio F. Espinoza Barría	University of Desarrollo, Faculty of Psychology, Santiago, Chile	mespinozab@udd.cl
Merve A. Kurfali	Bilkent University, Department of Political Science, Ankara, Turkey	merve.akdemir@bilkent.edu.tr
Michael C Mensink	University of Wisconsin-Stout, Department of Psychology, Menomonie, United States	mensinkm@uwstout.edu
Mikayel Harutyunyan	Charles University, Institute of Economic Studies, Prague, Czechia	75686400@fsv.cuni.cz
Meetu Khosla	University of Delhi, Psychology Department, DRC, Delhi, India	meetukhosla@yahoo.co.in
Megan R. Dunn	Illinois Institute of Technology, Chicago, USA	mdunn2@hawk.iit.edu
Max Korbmacher	University of Bergen, Faculty of Psychology, Department of Biological and Medical Psychology, Bergen, Norway<br>Western Norway University of Applied Sciences, Department of Health and Functioning, Bergen, Norway	max.korbmacher@gmail.com
Matúš Adamkovič	University of Presov, Institute of Psychology, Faculty of Arts, Presov, Slovakia<br>CSPS Slovak Academy of Sciences, Institute of Social Sciences, Slovakia	matho.adamkovic@gmail.com
Matheus Fernando Felix Ribeiro	University of Brasilia, Institute of Psychology, Brasilia, Brazil	matheusfelix.psi@gmail.com
Maria Terskova	HSE University, Moscow, Russia	materskova@gmail.com
Matej Hruška	Comenius University in Bratislava, Institute of European Studies and International Relations, Faculty of Social and Economic Sciences, Bratislava, Slovakia	matej.hruska@fses.uniba.sk
Marcel Martončik	University of Presov, Faculty of Arts, Presov, Slovakia; Institute of Social Sciences CSPS SAS, Slovakia	martoncik@protonmail.ch
Martine Jansen	Fontys University of Applied Sciences, Eindhoven, Netherlands	martine.jansen@gmail.com

Martin Voracek	University of Vienna, Department of Cognition, Emotion, and Methods in Psychology, Faculty of Psychology, Vienna, Austria	martin.voracek@univie.ac.at
Martin Čadek	Leeds Beckett University, Carnegie School of Sport, Leeds, United Kingdom	cadekmail@gmail.com
Martha Frias-Armenta	Universidad de Sonora, Sonora, Mexico	martha.frias@unison.mx
Marta Kowal	University of Wroclaw, Institute of Psychology, Wroclaw, Poland	marta7kowal@gmail.com
Marta Topor	University of Surrey, School of Psychology, Guildford, United Kingdom	m.topor@surrey.ac.uk
Marta Roczniewska	SWPS University of Social Sciences and Humanities in Sopot, Department of Psychology, Sopot, Poland<br>Karolinska Institutet, Department of Learning, Informatics, Management, and Ethics, Stockholm, Sweden	marta.roczniewska@swps.edu.pl
Marlies Oosterlinck	Independent Researcher	marliesoosterlinck@gmail.com
Markéta Braun Kohlová	Charles University, Environment Centre, Czechia	marketa.braun.kohlova@czp.cuni.cz
Mariola Paruzel-Czachura	Institute of Psychology, University of Silesia in Katowice, Poland & Faculty of Psychology, Complutense University of Madrid, Spain	mariola.paruzel-czachura@us.edu.pl
Marina Sabristov	Independent Researcher	marina.sabristov@gmail.com
Marina Romanova	HSE University, Moscow, Russia	marina.romanova.msk@gmail.com
Marietta Papadatou-Pastou	National and Kapodistrian University of Athens, Athens, Greece	marietta.papadatou-pastou@seh.oxon.org
Maria Louise Lund	University of Oslo, Oslo, Norway	marialouiselund@hotmail.com
Maria Antoniadi	University of Cyprus, Nicosia, Cyprus	maria.antoniadi867@gmail.com
Maria Elena Magrin	University of Milano – Bicocca, Italy	mariaelena.magrin@unimib.it
Marc V Jones	Manchester Metropolitan University, Department of Psychology, Manchester, United Kingdom	marc.jones@mmu.ac.uk
Manyu Li	University of Louisiana at Lafayette, Lafayette, United States	manyu.li@louisiana.edu
Manuel S Ortiz	Universidad de La Frontera, Departamento de Psicología.	manuel.ortiz@ufrontera.cl

	Laboratorio de Estrés y Salud, Temuco, Chile	
Mathi Manavalan	University of Minnesota, Twin Cities, Department of Psychology, Minneapolis, United States	manav003@umn.edu
Abdumalik Muminov	Independent Researcher	malik.traductor@gmail.com
Małgorzata Kossowska	Jagiellonian University, Department of Philosophy, Institute of Psychology, Krakow, Poland	malgorzata.kossowska@uj.edu.pl
Maja Friedemann	University of Oxford, Oxford, United Kingdom	maja.friedemann@sjc.ox.ac.uk
Magdalena Wielgus	Jagiellonian University, Institute of Applied Psychology, Krakow, Poland	magda.wielgus@uj.edu.pl
Madelon L.M. van Hooff	Radboud University, Behavioural Science Institute, Nijmegen, the Netherlands	madelon.vanhooff@ru.nl
Marco A. C. Varella	University of São Paulo, Institute of Psychology, Department of Experimental Psychology, São Paulo, Brazil	macvarella@usp.br
Martyn Standage	University of Bath, Centre for Motivation and Health Behaviour Change, Department for Health, United Kingdom	m.standage@bath.ac.uk
Matilde Nicolotti	University of Milano-Bicocca, Department of Psychology, Milan, Italy	m.nicolotti@campus.unimib.it
Melissa F Colloff	University of Birmingham, Birmingham, United Kingdom	m.colloff@bham.ac.uk
Maria Bradford	Universidad de los Andes, Department of Psychology, Bogotá, Colombia	m.bradford10@uniandes.edu.co
Leigh Ann Vaughn	Ithaca College, Ithaca, NY, United States	Lvaughn@ithaca.edu
Luis Eudave	Universidad de Navarra, Pamplona, Spain	luiseudave@gmail.com
Luc Vieira	Université de Paris, Paris, France	lucvieira@protonmail.com
Jackson G. Lu	Massachusetts Institute of Technology, Cambridge, United States	lu18@mit.edu
Lina Maria Sanabria Pineda	Universidad de los Andes, Department of Psychology, Bogotá, Colombia	lsanabriapineda@gmail.com

Lennia Matos	Pontifical Catholic University of Peru, Lima, Peru	lmatosf@pucp.pe
Laura Calderón Pérez	Universidad de los Andes, Department of Psychology, Bogotá, Colombia	lm.calderon10@uniandes.edu.co
Ljiljana B. Lazarevic	University of Belgrade, Faculty of Philosophy, Belgrade, Serbia	ljiljana.lazarevic@f.bg.ac.rs
Lisa M Jaremka	University of Delaware, Department of Psychological and Brain Sciences, Newark, United States	ljaremka@udel.edu
Eline Suzanne Smit	University of Amsterdam/ASCoR, Amsterdam, The Netherlands	E.S.Smit@uva.nl
Elizaveta Kushnir	Independent Researcher	lizakushnir@yandex.ru
Lisa J. Ferguson	Northumbria University, Newcastle upon Tyne, United Kingdom	lisa2.ferguson@northumbria.ac.uk
Lisa Anton-Boicuk	University of Vienna, Social, Cognitive and Affective Neuroscience Unit, Department of Cognition, Emotion, and Methods in Psychology, Faculty of Psychology, Vienna, Austria	lisa.anton-boicuk@univie.ac.at
Gabriel Lins de Holanda Coelho	University College Cork, Cork, Ireland	linshc@gmail.com
Lina Ahlgren	Åbo Akademi University, Faculty of Arts, Psychology and Theology, Turku, Finland	lina.ahlgren@gmail.com
Francesca Liga	Università di Messina, Dipartimento DIMED, Messina, Italy	ligaf@unime.it
Carmel A Levitan	Occidental College, Department of Cognitive Science, Los Angeles, United States	levitan@oxy.edu
Leticia Micheli	Julius-Maximilians Universität Würzburg, Institute of Psychology, Würzburg, Germany	leticia.micheli@uni-wuerzburg.de
Lesley-Ann Gunton	Northumbria University, Newcastle upon Tyne, United Kingdom	lesley-ann.gunton@northumbria.ac.uk
Leonhard Volz	University of Amsterdam, Amsterdam, Netherlands	leonhard.volz@gmail.com
Marija Stojanovska	PSA Psihesko, Skopje, North Macedonia	lemarija22@gmail.com
Leanne Boucher	Nova Southeastern University, Department of Psychology and Neuroscience, Fort Lauderdale, United States	lb1079@nova.edu

Lara Samojlenko	University of Primorska, Department of Psychology, Faculty of Mathematics, Natural Sciences and Information Technologies, Koper, Slovenia	lara.samojlenko@gmail.com
Lady Grey Javela Delgado	Universidad del Rosario, Programa de Psicología, Bogotá, Colombia	lady.javela@urosario.edu.co
Lada Kaliska	Matej Bel University, Department of Psychology, Faculty of Education, Banska Bystrica, Slovakia	lada.kaliska@umb.sk
Labadi Beatrix	University of Pécs, Institute of Psychology, Pécs, Hungary	labadi.beatrix@pte.hu
Lara Warmelink	Lancaster University, Department of Psychology, Lancaster, United Kingdom	l.warmelink@lancaster.ac.uk
Luis Miguel Rojas-Berscia	University of Queensland, School of Languages and Cultures, Brisbane, Australia<br>Pontificia Universidad Católica del Perú, Centro de Estudios Orientales, Lima, Peru	lmrojasb@pucp.pe
Karen Yu	Sewanee: The University of the South, Department of Psychology, Sewanee, TN, United States	kyu@sewanee.edu
Keith Wylie	Emporia State University, Department of Psychology, Emporia, Kansas, United States	kwylie@emporia.edu
Jakub Wachowicz	Independent Researcher	kubawachowicz7@gmail.com
Kermeka Desai	Indiana University of Pennsylvania, Indiana, United States	kermeka@gmail.com
Krystian Barzykowski	Jagiellonian University, Institute of Psychology, Krakow, Poland	krystian.barzykowski@uj.edu.pl
Luca Kozma	University of Pécs, Institute of Psychology, Pécs, Hungary<br>University of the West of Scotland, School of Education and Social Sciences, Division of Psychology, Paisley, UK	luca.kozma@uws.ac.uk
Kortnee Evans	Murdoch University, College of Science, Health, Engineering and Education, Perth, Australia	kortnee.evans@education.wa.edu.au
Komila Kirgizova	Independent Researcher	komila@hotmail.it
Bamikole B Emmanuel Agesin	Adekunle Ajasin University, Akungba Akoko, Ondo State, Nigeria	koleagesin@yahoo.com

Monica A Koehn	University of Canberra, Discipline of Psychology, Faculty of Health, Canberra, Australia	koehn.monica@gmail.com
Kelly Wolfe	University of Edinburgh, Edinburgh, United Kingdom	kwolfe@ed.ac.uk
Tatiana Korobova	London Gates Education Group, Moscow, Russia	klushca@gmail.com
Katherine Morris	Willamette University, Salem, United States	klmorris249@gmail.com
Kristoffer Klevjer	UiT The Arctic University of Norway, Department of Psychology, Tromsø, Norway	klevjer@gmail.com
Kevin van Schie	Erasmus University Rotterdam, Department of Psychology, Education & Child Studies, Erasmus School of Social and Behavioural Sciences, Rotterdam, Netherlands<br>University of Cambridge, MRC Cognition and Brain Sciences Unit, Cambridge, United Kingdom	kevinvschie@gmail.com
Kevin Vezirian	University of Grenoble Alpes, Grenoble, France	kevin.vezirian@gmail.com
Kaja Damnjanović	University of Belgrade, Faculty of Philosophy, Belgrade, Serbia	kdamnjan@f.bg.ac.rs
Katrine Krabbe Thommesen	University of St Andrews, School of Psychology and Neuroscience, Zealand, Denmark	katrinekrabbe@gmail.com
Kathleen Schmidt	Southern Illinois University, School of Psychological and Behavioral Sciences, Carbondale, IL United States	kathleen.schmidt@siu.edu
Katarzyna Filip	Jagiellonian University, Institute of Psychology, Krakow, Poland	katarzyna.filip95@gmail.com
Karolina Staniaszek	Independent Researcher	karolina.staniaszek@gmail.com
Karolina Grzech	University of Valencia, Valencia, Spain<br>Stockholm University, Stockholm, Sweden	szarota@gmail.com
Karlijn Hoyer	Tilburg University, Tilburg, Netherlands	karlijnhoyer@gmail.com
Karis Moon	Kingston University London, Department of Management, Kingston, United Kingdom	Karisamoon@gmail.com

Sirikon Khaobunmasiri	Suranaree University of Technology, Nakhon Ratchasima, Thailand	kanjana@sut.ac.th
Kafeel Rana	GC University, Lahore, Pakistan	kafeelrana87@gmail.com
Kristina Janjić	PSA Psihesko, Skopje, North Macedonia	k.janjic@yahoo.com
Jordan W Suchow	Stevens Institute of Technology, School of Business, Hoboken, United States	jws@stevens.edu
Julita Kielińska	Jagiellonian University, Institute of Psychology, Krakow, Poland	julita.kielinska@alumni.uj.edu.pl
Julio E Cruz Vásquez	Universidad de los Andes, Department of Psychology Bogotá, Colombia	julioeduardocruz@gmail.com
Julien Chanal	University of Geneva, Geneva, Switzerland	julien.chanal@unige.ch
Julia Beitner	Goethe University Frankfurt, Department of Psychology, Frankfurt am Main, Germany	beitner@psych.uni-frankfurt.de
Juan Camilo Vargas-Nieto	Universidad de los Andes, Department of Psychology, Bogotá, Colombia	juanvargaspsicologia@gmail.com
Jose Carlos T Roxas	University of the Philippines Diliman, Department of Psychology, Quezon City, Philippines<br>De La Salle College of Saint Benilde, Department of Psychology, Antipolo, Philippines	jtroxas@up.edu.ph
Jennifer Taber	Kent State University, Department of Psychological Sciences, Kent, United States	jtaber1@kent.edu
Joan Urriago-Rayo	Independent Researcher	joan.urriago.rayo@gmail.com
Jeffrey M. Pavlacic	University of Mississippi, Department of Psychology, Oxford, Mississippi, United States	jpavlaci@go.olemiss.edu
Jozef Benka	Pavol Jozef Šafárik University, Košice, Slovakia	jozef.benka@upjs.sk
Jozef Bavolar	Pavol Jozef Šafárik University in Košice, Department of Psychology, Faculty of Arts, Košice, Slovakia	jozef.bavolar@upjs.sk
José A. Soto	The Pennsylvania State University, Department of Psychology, State College, United States	josesoto@psu.edu
Jonas K Olofsson	Stockholm University, Department of Psychology, Stockholm, Sweden	jonas.olofsson@psychology.su.se

Johannes K Vilsmeier	University of Vienna, Department of Cognition, Emotion, and Methods in Psychology, Vienna, Austria	johannes.vilsmeier@univie.ac.at
Johanna Messerschmidt	Leipzig University, Institute of Psychology, Leipzig, Germany	johanna.messerschmidt@gmail.com
Johanna Czamanski-Cohen	University of Haifa, School of Creative Arts Therapies, Haifa, Israel University of Haifa, Emili Sagol Creative Arts Therapies Research Center, Haifa, Israel	joczamanski@gmail.com
Joachim Waterschoot	Ghent University, Department of Developmental, Personality and Social Psychology, Ghent, Belgium	joachim.waterschoot@ugent.be
Jennifer D. Moss	Emporia State University, Department of Psychology, Emporia, Kansas, United States	jmoss3@emporia.edu
Jordane Boudesseul	Universidad de Lima, Facultad de Psicología, Instituto de Investigación Científica, Lima, Peru	jmj.boudesseul@gmail.com
Jeong Min Lee	Georgia State University, Department of Psychology, Atlanta, United States	jlee500@gsu.edu
Julia Kamburidis	Sofia University St. Kliment Ohridski, Department of General, Experimental, Developmental, and Health Psychology, Sofia, Bulgaria	jkamburidis@gmail.com
Jennifer A Joy-Gaba	Virginia Commonwealth University, Richmond, United States	jjoygaba@vcu.edu
Janis Zickfeld	Aarhus University, Department of Management, Aarhus, Denmark	jhzickfeld@gmail.com
Jacob F Miranda	The University of Alabama, Tuscaloosa, Department of Psychology, Tuscaloosa, United States	jfmiranda@crimson.ua.edu
Jeroen P.H. Verharen	University of California Berkeley, Department of Molecular and Cell Biology, Berkeley, United States	jeroenverharen@berkeley.edu
Evgeniya Hristova	New Bulgarian University, Cognitive Science and Psychology Department, Sofia, Bulgaria	ehristova@cogs.nbu.bg
Julie E Beshears	University of Southern Indiana, Evansville, United States	jeb1118@comcast.net
Jasna Milosevic Djordjevic	Singidunum University, Faculty of Media and Communication, Belgrade, Serbia	jasna.milosevic@yahoo.com

Jasmijn Bosch	University of Milan-Bicocca, Milan, Italy	Jasmijn.e.bosch@gmail.com
Jaroslava Varella Valentova	University of São Paulo, Department of Experimental Psychology, Institute of Psychology, São Paulo, Brazil	jaroslava@usp.br
Jan Antfolk	Åbo Akademi University, Faculty of Arts, Psychology and Theology, Turku, Finland	jantfolk@abo.fi
Jana B. Berkessel	University of Mannheim, Mannheim Centre for European Social Research, Mannheim, Germany	jana.berkessel@uni-mannheim.de
Jana Schrötter	Pavol Jozef Šafárik University in Košice, Košice, Slovakia	jana.schrotter@upjs.sk
Jan Urban	Charles University, Environment Centre, Czechia	jan.urban@czp.cuni.cz
Jan Philipp Röer	Witten/Herdecke University, Department of Psychology, Witten, Germany	jan.roeer@uni-wh.de
James O Norton	Murdoch University, College of Science, Health, Engineering & Education, Perth, Australia	james.norton@murdoch.edu.au
Jaime R Silva	University of Desarrollo, Faculty of Psychology, Santiago, Chile<br>Clínica Alemana de Santiago, Chile<br>Sociedad Chilena de Desarrollo Emocional, Chile	jaimesilva@udd.cl
Jade S Pickering	University of Manchester, Division of Neuroscience and Experimental Psychology, Manchester, United Kingdom	jadespickering@gmail.com
Jáchym Vintr	Charles University, Department of Psychology, Faculty of Arts, Prague, Czechia	vintrj@student.cuni.cz
Jim Uttley	University of Sheffield, School of Architecture, Sheffield, United Kingdom	j.uttley@sheffield.ac.uk
Jonas R Kunst	University of Oslo, Department of Psychology, Oslo, Norway	j.r.kunst@psykologi.uio.no
Izuchukwu L. G. Ndukaihe	Alex Ekwueme Federal University, Department of Psychology, Ndufu-Alike, Nigeria.	izumario@yahoo.co.uk
Aishwarya Iyer	Sampurna Montfort College, Bangalore, India	iyeraishwarya.work@gmail.com

Iris Vilares	University of Minnesota, Twin Cities, Department of Psychology, Minneapolis, United States	ivilares@umn.edu
Aleksandr Ivanov	HSE University, Moscow, Russia	ivansash21112@mail.ru
Ivan Ropovik	Charles University, Faculty of Education, Institute for Research and Development of Education, Prague, Czechia<br>University of Presov, Faculty of Education, Presov, Slovakia	ivan.ropovik@gmail.com
Isabela Sula	Independent Researcher	isabela.sula1997@gmail.com
Irena Sarieva	HSE University, Moscow, Russia	isarieva@hse.ru
Irem Metin-Orta	Atilim University, Department of Psychology, Ankara, Turkey	irem.metin@atilim.edu.tr
Irina Prusova	HSE University, Moscow, Russia	iprusova@hse.ru
Isabel Pinto	University of Porto, Center for Psychology at University of Porto, Porto, Portugal	ipinto@fpce.up.pt
Andreea Ioana Bozdoc	Lucian Blaga University of Sibiu, Department of Psychology, Sibiu, Romania	ioanabozdoc@gmail.com
Inês A. T. Almeida	University of Coimbra, Faculty of Medicine FMUC, Institute of Nuclear Sciences Applied to Health ICNAS, Coimbra Institute for Biomedical Imaging and Translational Research CIBIT, Coimbra, Portugal	italmeida@fmed.uc.pt
Ilse L. Pit	University of Oxford, Institute of Human Sciences, Oxford, United Kingdom<br>Magdalen College, Calleva Research Centre for Evolution and Human Sciences, Oxford, United Kingdom	ilse.pit@anthro.ox.ac.uk
Ilker Dalgar	Ankara Medipol University, Department of Psychology, Ankara, Turkey	ilker.dalgar@ankaramedipol.edu.tr
Ilya Zakharov	Psychological Institute of the Russian Academy of Education, Developmental Behavioral Genetics Laboratory, Moscow, Russia, Russia	iliazaharov@gmail.com
Azuka Ikechukwu Arinze	Alex Ekwueme Federal University, Ndufu-Alike, Nigeria	ikeazukaarinze@gmail.com
Keiko Ihaya	Fukuoka Institute of Technology, Center for Liberal Arts, Fukuoka, Japan	ihayakk@gmail.com

Ian D Stephen	Nottingham Trent University, Division of Psychology, Nottingham, UK	ian.stephen@ntu.ac.uk
Biljana Gjoneska	Macedonian Academy of Sciences and Arts, Skopje, North Macedonia	biljanagjoneska@manu.edu.mk
Hilmar Brohmer	University of Graz, Institute of Psychology, Graz, Austria	hilmar.brohmer@uni-graz.at
Heather Flowe	University of Birmingham, School of Psychology, Birmingham, United Kingdom	h.flowe@bham.ac.uk
Hendrik Godbersen	FOM University of Applied Sciences, Essen, Germany	hendrik.godbersen@godbersen.onl ine
Halil Emre Kocalar	Muğla Sıtkı Koçman University, Department of Psychological Counseling and Guidance, Muğla, Turkey	hemrekocalar@mu.edu.tr
Mattie V Hedgebeth	Virginia Commonwealth University, Richmond, United States	hedgebethm@vcu.edu
Hu Chuan-Peng	Nanjing Normal University, School of Psychology, Nanjing, China	hcp4715@gmail.com
MohammadHasan Sharifian	University of Tehran, Department of Psychology, Tehran, Iran	hasan.sharifian@ut.ac.ir
Harry Manley	Chulalongkorn University, Faculty of Psychology, , Bangkok, Thailand	harrisonmanley@gmail.com
Handan Akkas	Ankara Science University, Business Administration Department, Ankara, Turkey	handan.akkas@hotmail.com
Nandor Hajdu	ELTE Eötvös Lóránd University, Institute of Psychology, Budapest, Hungary	hajdu.nandor93@gmail.com
Habiba Azab	Baylor College of Medicine, Department of Neurosurgery, Houston, United States	habiba.azab@gmail.com
Gwenael Kaminski	Université de Toulouse, CLLE, CNRS, UT2J, Toulouse, France	gwenael.kaminski@univ-tlse2.fr
Gustav Nilsonne	Karolinska Institutet, Department of Clinical Neuroscience, Solna, Sweden Stockholm University, Department of Psychology, Stockholm, Sweden	gustav.nilsonne@ki.se
Gulnaz Anjum	Simon Fraser University, Department of Psychology, Burnaby, Canada Institute of Business Administration, Department of Social Sciences & Liberal Arts, Karachi, Pakistan	ganjum@sfu.ca

Giovanni A. Travaglino	Royal Holloway, University of London, Department of Law and Criminology, Egham, United Kingdom	giovanni.travaglino@rhul.ac.uk
Gilad Feldman	University of Hong Kong, Hong Kong, SAR China	giladfel@gmail.com
Gerit Pfuhl	UiT The Arctic University of Norway, Department of Psychology, Tromsø, Norway	gerit.pfuhl@uit.no
Gabriela Czarnek	Jagiellonian University, Institute of Psychology, Krakow, Poland	gabriela.czarnek@uj.edu.pl
Gabriela Mariana Marcu	Lucian Blaga University of Sibiu, Department of Psychology, Sibiu Romania<br>Carol Davila University of Medicine and Pharmacy Bucharest, Romania	gabriela.marcu@ulbsibiu.ro
Gabriela Hofer	University of Graz, Institute of Psychology, Graz, Austria	gabriela.hofer@uni-graz.at
Gabriel Banik	University of Presov, Institute of Psychology, Presov, Slovakia	gabriel.banik@gmail.com
Gabriel Agboola Adetula	Adekunle Ajasin University, Department of Pure and Applied Psychology, Faculty of Social and Management Sciences, Akungba Akoko, Nigeria	g1b2gbo3detul4@gmail.com
Gijsbert Bijlstra	Radboud University, Behavioural Science Institute, Nijmegen, Netherlands	g.bijlstra@bsi.ru.nl
Frederick Verbruggen	Ghent University, Department of Experimental Psychology, Ghent, Belgium	frederick.verbruggen@ugent.be
Franki Y. H. Kung	Purdue University, West Lafayette, United States	frankikung@purdue.edu
Frank Martela	Aalto University, Espoo, Finland	frank.martela@aalto.fi
Francesco Foroni	Australian Catholic University, Sydney, Australia	francesco.foroni@acu.edu.au
Jacques Forest	Université du Québec à Montréal, School of Management, Montreal, Canada	forest.jacques@uqam.ca
Gage Singer	Indiana University of Pennsylvania, Department of Psychology, Indiana, United States	gagesinger@live.com

Fany Muchembled	Instituto Tecnológico de Estudios Superiores de Monterrey, Monterrey, Mexico	fany.muchembled@tec.mx
Flavio Azevedo	Friedrich Schiller University Jena, Jena, Germany	flavio.azevedo@uni-jena.de
Farnaz Mosannenzadeh	Radboud University, Faculty of Social Sciences, Behavioural Science Institute, Nijmegen, Netherlands	farnaz.mosannenzadeh@ru.nl
Evelina Marinova	Sofia University St. Kliment Ohridski, Department of General, Experimental, Developmental, and Health Psychology, Sofia, Bulgaria	evelina.b.marinova@gmail.com
Eva Štrukelj	Sapienza University of Rome, Dynamic and Clinical Psychology, Rome, Italy	eva.strukelj2@gmail.com
Zahra Etebari	Ferdowsi University of Mashhad, Mashhad, Iran	etebari.zahra@gmail.com
Emma L. Bradshaw	Australian Catholic University, Institute for Positive Psychology and Education, Sydney, Australia	emma.bradshaw@acu.edu.au
Ernest Baskin	Saint Joseph's University, Philadelphia, United States	ebaskin@sju.edu
Elkin Oswaldo Luis Garcia	Universidad de Navarra, Pamplona, Spain	eoswaldo@unav.es
Erica Musser	Florida International University, Department of Psychology, Center for Children and Families, Miami, United States	emusser@fiu.edu
I.M.M. van Steenkiste	Universiteit Leiden, Leiden, Netherlands	imvsteenkiste@gmail.com
El Rim Ahn	University of Florida, Department of Psychology, Gainesville, United States	elrimahn@ufl.edu
Eleanor Quested	Curtin University, enAble Institute, Perth, Australia	eleanor.quested@curtin.edu.au
Ekaterina Pronizius	University of Vienna, Department of Cognition, Emotion, and Methods in Psychology, Faculty of Psychology, Vienna, Austria	ekaterina.pronizius@univie.ac.at
Emily A Jackson	Indiana University of Pennsylvania, Indiana, United States	ejackson@iup.edu
Efisio Manunta	Université de Toulouse, CLLE, CNRS, UT2J, Toulouse, France	efisio.manunta@univ-tlse2.fr

Elena Agadullina	HSE University, School of Psychology, Moscow, Russia	eagadullina@hse.ru
Dušana Šakan	Union University, Faculty of Legal and Business Studies Dr Lazar Vrkatic, Department of Psychology, Novi Sad, Serbia	dusana.sakan@flv.edu.rs
Pinar Dursun	Afyon Kocatepe University, Department of Psychology, Afyonkarahisar, Turkey	dursun.pinar@gmail.com
Olivier Dujols	University of Grenoble Alpes, Grenoble, France	dujols.ol@gmail.com
Dmitrii Dubrov	National Research University Higher School of Economics, RF	ddubrov@hse.ru
Megan Willis	Australian Catholic University, School of Behavioural and Health Sciences, Sydney, Australia	Dr.Megan.Willis@gmail.com
Murat Tümer	Hacettepe University, Department of Anesthesiology and Reanimation, Ankara, Turkey	m.tumer@hacettepe.edu.tr
Jennifer L Beaudry	Swinburne University of Technology, Department of Psychological Sciences, Melbourne, Australia	jbeaudry@swin.edu.au
Dora Popović	Institute of Social Sciences Ivo Pilar, Zagreb, Croatia	dora.popovic@pilar.hr
Daniel Dunleavy	Florida State University, Center for Translational Behavioral Science, Tallahassee, United States	djd09e@fsu.edu
Ikhlas Djamai	Mohammed V University in Rabat, Rabat, Morocco	djamaiikhlas@gmail.com
Dino Krupić	The University of Osijek, Faculty of Humanities and Social Science, Osijek, Croatia	dkrupic@ffos.hr
Dora Herrera	Pontifical Catholic University of Peru, Lima, Peru	diherrer@pucp.pe
Diego Vega	Universidad Latina de Costa Rica, San Pedro, Costa Rica	luis.vegaa@ulatina.cr
Hongfei Du	Beijing Normal University at Zhuhai, Institute of Advanced Studies in Humanities and Social Sciences, Zhuhai, China	dhfpsy@gmail.com
Débora Mola	Universidad Nacional de Córdoba (UNC), Facultad de Psicología; Instituto de Investigaciones Psicológicas (IIPsi) - Consejo	debora.mola@unc.edu.ar

	Nacional de Investigaciones Científicas y Técnicas (CONICET) - UNC, Córdoba, Argentina	
Desislava Chakarova	New Bulgarian University, Sofia, Bulgaria	de.chakarova@gmail.com
William E Davis	Wittenberg University, Department of Psychology, Springfield, United States	davisw4@wittenberg.edu
Dawn Liu Holford	University of Essex, Essex, United Kingdom	dawn.liuholford@gmail.com
David M. G. Lewis	Murdoch University, College of Science, Health, Engineering and Education, Perth, Australia; Murdoch University, Centre for Healthy Ageing, Health Futures Institute, Perth, Australia,	davidlewis@utexas.edu
David C. Vaidis	Université de Paris, Paris, France	david.vaidis@u-paris.fr
Daphna Hausman Ozery	California State University, Northridge, United States	daphna.ozery@csun.edu
Danilo Zambrano Ricaurte	Fundación Universitaria Konrad Lorenz, Faculty of Psychology, Bogotá, Colombia	danilo.zambranor@konradlorenz.edu.co
Daniel Storage	University of Denver, Department of Psychology, Denver, United States	Daniel.Storage@du.edu
Daniela Sousa	University of Coimbra, Institute of Nuclear Sciences Applied to Health ICNAS, Coimbra Institute for Biomedical Imaging and Translational Research CIBIT, Coimbra, Portugal	daniela.d.sousa@uc.pt
Daniela Serrato Alvarez	Fundación Universitaria Konrad Lorenz, Bogotá, Colombia	daniela.serratoa@konradlorenz.edu.co
Daniel Boller	University of St. Gallen, St. Gallen, Switzerland	daniel.boller@unisg.ch
Anna Dalla Rosa	University of Padova, Department of Philosophy, Sociology, Education and Applied Psychology, Padua, Italy	anna.dallarosa@unipd.it
Daliborka Dimova	PSA Psihesko, Skopje, North Macedonia	daliborkadimova@gmail.com
Dajana Krupić	Norvel - Psychological Centre for Counselling and Research, Croatia	dajana.krupic@norvel.hr
Dafne Marko	University of Ljubljana, Cognitive Science, Faculty of Education, Ljubljana, Slovenia	dafne.marko@gmail.com

David Moreau	The University of Auckland, School of Psychology and Centre for Brain Research, Auckland, New Zealand	d.moreau@auckland.ac.nz
Crystal Reeck	Temple University, Fox School of Business, Philadelphia, United States	crystalreeck@gmail.com
Rita C Correia	University of Porto, Center for Psychology at University of Porto, Porto, Portugal	correia.rita.27@gmail.com
Cassie M Whitt	University of Alabama, Tuscaloosa, United States	cassiewhitt9@gmail.com
Claus Lamm	University of Vienna, Department of Cognition, Emotion, and Methods in Psychology, Faculty of Psychology, Vienna, Austria	claus.lamm@univie.ac.at
Claudio Singh Solorzano	Sapienza University, Department of Psychology, Rome, Italy	claudio.singh@uniroma1.it
Claudia C von Bastian	University of Sheffield, Department of Psychology, Sheffield, United Kingdom	c.c.vonbastian@sheffield.ac.uk
Clare AM Sutherland	University of Aberdeen, School of Psychology, King's College, Aberdeen, Scotland<br><br>University of Western Australia, School of Psychological Science, Perth, Australia	clare.sutherland@abdn.ac.uk
Clara Overkott	University of Zurich, Department of Psychology, Zurich, Switzerland	c.overkott@psychologie.uzh.ch
Christopher L. Aberson	California Polytechnic University, Humboldt, Arcata, United States	cla18@humboldt.edu
Chunhui Wang	Chinese Center of Disease Prevention and Control, China	chunhui.wang.qdjk@gmail.com
Christopher P. Niemiec	University of Rochester, Rochester, United States	christopher.niemiec@rochester.edu
Christiana Karashiali	University of Cyprus, Department of Psychology, Nicosia, Cyprus	karashiali.christiana@ucy.ac.cy
Chris Noone	National University of Ireland Galway, School of Psychology, Galway, Ireland	chris.noone@nuigalway.ie
Faith Chiu	University of Essex, Department of Language and Linguistics, Essex, United Kingdom	f.chiu@essex.ac.uk
Chiara Picciocchi	University of Naples L'Orientale, Naples, Italy	chiara.picciocchi@outlook.com

Charlotte Brownlow	University of Southern Queensland, Toowoomba, Australia	Charlotte.brownlow@usq.edu.au
Cemre Karaarslan	University of Başkent, Institute of Social Sciences, Department of Psychology, Ankara, Turkey	cemrekaraarslann@gmail.com
Nicola Cellini	University of Padua, Department of General Psychology, Padua, Italy<br>University of Padua, Department of Biomedical Sciences, Padua, Italy<br>University of Padua, Padova Neuroscience Center, Padua, Italy<br>University of Padua, Human Inspired Technology Center, Padua, Italy	nicola.cellini@unipd.it
Celia Esteban-Serna	University College London, Division of Psychology & Language Sciences, London, United Kingdom	celiaestser99@gmail.com
Cecilia Reyna	Universidad Nacional de Córdoba (UNC), Facultad de Psicología; Instituto de Investigaciones Psicológicas (IIPsi) - Consejo Nacional de Investigaciones Científicas y Técnicas (CONICET) - UNC, Córdoba, Argentina	ceciliareyna@unc.edu.ar
Cecilia Ferreyra	Pontificia Universidad Católica del Peru, Lima, Peru	cecilia.ferreyra@pucp.pe
Carlota Batres	Franklin and Marshall College, Department of Psychology, Lancaster, United States	cbatres@fandm.edu
Ranran Li	Vrije Universiteit Amsterdam, Department of Experimental and Applied Psychology, Amsterdam, Netherlands	ranran.li@vu.nl
Caterina Grano	Sapienza University, Department of Psychology, Rome, Italy	caterina.grano@uniroma1.it
Joelle Carpentier	Université du Québec à Montréal, School of Management, Department of Organization and Human Resources, Montreal, Canada	carpentier.joelle@uqam.ca
Christian K. Tamnes	University of Oslo, Department of Psychology, Oslo, Norway	c.k.tamnes@psykologi.uio.no
Cynthia H.Y. Fu	University of East London, School of Psychology, London, United Kingdom<br>King's College London, Centre for Affective Disorders, Institute of Psychiatry, Psychology and Neuroscience, London, United Kingdom	c.fu@uel.ac.uk

Byurakn Ishkhanyan	Aarhus University, School of Communication and Culture, Aarhus, Denmark	byurakn@cc.au.dk
Lisa Bylinina	Leiden University, Leiden, Netherlands	bylinina@gmail.com
Bastian Jaeger	Vrije Universiteit Amsterdam, Department of Experimental and Applied Psychology, Amsterdam, Netherlands<br>Tilburg University, Department of Social Psychology, Tilburg, Netherlands	bxjaeger@gmail.com
Carsten Bundt	Multimodal Imaging and Cognitive Control Lab, Department of Psychology, University of Oslo, Oslo, Norway<br>Cognitive and Translational Neuroscience Cluster, Department of Psychology, University of Oslo, Oslo, Norway	bundt.carsten@gmail.com
Tara Bulut Allred	University of Belgrade, Faculty of Philosophy, Laboratory for Research of Individual Differences , Belgrade, Serbia	tara.bulut@f.bg.ac.rs
Branko J. Vermote	Ghent University, Department of Developmental, Personality and Social Psychology, Ghent, Belgium	branko.vermote@ugent.be
Ahmed Bokkour	Mohammed V University in Rabat, Rabat, Morocco	bokkour.ahmed@gmail.com
Natalia Bogatyreva	HSE University, Moscow, Russia	nbogatyreva@hse.ru
Jiaxin Shi	The University of Hong Kong, Hong Kong, SAR China	langlang723@foxmail.com
William J Chopik	Michigan State University, Department of Psychology, East Lansing, United States	bill.chopik@gmail.com
Benedict Antazo	Jose Rizal University, Department of Psychology, Mandaluyong, Philippines	bgantazo@gmail.com
Behzad Behzadnia	University of Tabriz, *Faculty of Physical Education and Sport Science*, Department of Motor Behavior, Tabriz, Iran	behzadniaa@gmail.com
Maja Becker	Université de Toulouse, CLLE, CNRS, Toulouse, France	mbecker@univ-tlse2.fr
Manal M. Bayyat	School of Sport Science, University of Jordan, Jordan	Mabayyat@yahoo.com

Beatrice Cocco	Independent Researcher	beatricecocco094@gmail.com
Wei-Lun Chou	Fo Guang University, Department of Psychology, Jiaoxi, Taiwan	chouweilun@ntu.edu.tw
Vassilis Barkoukis	Department of Physical Education and Sport Science, Aristotle University of Thessaloniki, Greece	bark@phed.auth.gr
Barbora Hubena	Independent Researcher	barbora.hubena@gmail.com
Barbara Žuro	The Institute of Psychology, Dublin, Ireland<br>University of Osijek, Faculty of Humanities and Social Sciences, Osijek, Croatia	barbara.zuro1@gmail.com
Balazs Aczel	ELTE Eötvös Loránd University, Institute of Psychology, Budapest, Hungary	balazs.aczel@gmail.com
Ekaterina Baklanova	Lomonosov Moscow State University, Institute of Asian and African Studies, Moscow, Russia	baklanova@gmail.com
Hui Bai	Stanford University, Palo Alto, United States	huibai@stanford.edu
Busra Bahar Balci	Samsun University, Department of Psychology, Samsun, Turkey<br>Dokuz Eylül University, Department of Psychology, Izmir, Turkey	baharbalci2@gmail.com
Peter Babinčák	University of Presov, Faculty of Arts, Institute and Psychology, Presov, Slovakia	peter.babincak@unipo.sk
Bart Soenens	Department of Developmental, Personality and Social Psychology, Ghent University	Bart.Soenens@ugent.be
Barnaby James Wyld Dixson	University of the Sunshine Coast, School of Health and Behavioural Sciences, Sippy Downs, Australia	bdixson@usc.edu.au
Aviv Mokady	Ben Gurion University, Department of Psychology, Beersheba, Israel	avmokady@gmail.com
Heather Barry Kappes	London School of Economics and Political Science, Department of Management, London, United Kingdom	h.kappes@lse.ac.uk
Mohammad Atari	University of Southern California, Department of Psychology, Los Angeles, United States	atari@usc.edu
Anna Szala	Oakland University, Department of Psychology, Rochester, United States	aszala88@gmail.com

Anna Szabelska	Queen's University Belfast, Belfast, Ireland	szabelska.anna@gmail.com
John Jamir Benzon Aruta	De La Salle University, Manila, Philippines	aruta_johnjamirbenzon@yahoo.com
Artur Domurat	Kozminski University, Centre for Economic Psychology and Decision Sciences, Warsaw, Poland	adomurat@kozminski.edu.pl
Nwadiogo Chisom Arinze	Alex Ekwueme Federal University Ndufu-Alike, Nigeria	arinzenwadiogo@gmail.com
Arianna Modena	Università degli Studi di Trieste, Dipartimento di Scienze Giuridiche, del Linguaggio, dell'Interpretazione e della Traduzione, Trieste, Italy	ariannamodena95@gmail.com
Arca Adiguzel	Muğla Sıtkı Koçman University, Department of Psychological Counseling and Guidance, Muğla, Turkey	arcaadiguzel@mu.edu.tr
Arash Monajem	University of Tehran, Tehran, Iran	arash.monajem@hotmail.com
Kanza AIT EL ARABI	Mohammed V University of Rabat, Rabat, Morocco	arabikanza@gmail.com
Asil Ali Özdoğru	Üsküdar University, Department of Psychology, İstanbul, Turkey	asil.ozdogru@uskudar.edu.tr
Alex O. Rothbaum	Case Western Reserve University, Cleveland, United States	aor13@case.edu
Adriana Olaya Torres	University of Desarrollo, Faculty of Psychology, Santiago, Chile	aolayat@udd.cl
Andriana Theodoropoulou	University of Essex, Department of Psychology, Essex, United Kingdom	a.theodoropoulou@essex.ac.uk
Anna Skowronek	Independent Researcher	anna.allodola@gmail.com
Anita Penić Jurković	Kindergarten Kustošija, Zagreb, Croatia	anita.penic.vk@gmail.com
Anisha Singh	Busara Center of Behavioural Economics, Kenya	anisha.singh@busaracenter.org
Angelos P. Kassianos	Cyprus University of Technology, Department of Nursing, Limassol, Cyprus<br>University College London, Department of Applied Health Research, London, United Kingdom	angelos.kassianos@cut.ac.cy
Andrej Findor	Comenius University in Bratislava, Faculty of Social and Economic Sciences, Bratislava, Slovakia	andrej.findor@fses.uniba.sk
Javad Hatami	University of Tehran	hatamijm@ut.ac.ir

Andree Hartanto	Singapore Management University, School of Social Sciences, Singapore	andreeh@smu.edu.sg
Anais Thibault Landry	Concordia University, John Molson Business School, Montreal, Canada	Anais.thibaultlandry@gmail.com
Ana Ferreira	University of Coimbra, Faculty of Medicine FMUC, Institute of Nuclear Sciences Applied to Health ICNAS, Coimbra Institute for Biomedical Imaging and Translational Research CIBIT, Coimbra, Portugal	apferreira@icnas.uc.pt
Anabela Caetano Santos	University of Lisbon, Aventura Social and DESSH, Faculty of Human Kinetics,, Lisbon, Portugal<br>University of Lisbon, Institute of Environmental Health, Medicine Faculty, Lisbon, Portugal<br>Universitário de Lisboa, Iscte-Instituto Universitário de Lisboa, CIS-IUL, Lisbon, Portugal	anabelasantos@campus.ul.pt
Anabel De la Rosa-Gomez	National Autonomous University of Mexico, Faculty of Higher Studies Iztacala, Mexico City, Mexico	anabel.delarosa@iztacala.unam.mx
Amélie Gourdon-Kanhukamwe	Kingston University, London, United Kingdom<br>King's College London, London, United Kingdom<br>Institute for Globally Distributed Open Research and Education (IGDORE), United Kingdom	amelie.gourdon-kanhukamwe@kcl.ac.uk
Alexandria M. Luxon	Illinois Institute of Technology, Chicago, United States	aluxon@hawk.iit.edu
Anna Louise Todsen	University of St Andrews, Department of Psychology and Neuroscience, St Andrews, United Kingdom	alt8@st-andrews.ac.uk
Alper Karababa	Muğla Sıtkı Koçman University, Department of Psychological Counselling and Guidance, Faculty of Education, Muğla, Turkey	alperkarababa@mu.edu.tr
Allison Janak	New York University, Steinhardt, Department of Applied Psychology, New York, United States	apj263@nyu.edu
Alice Pilato	University of Trieste, Department of Translation and Interpretation, Trieste, Italy	alicepilato98@gmail.com
Alexandre Bran	Université de Paris, Paris, France	alexandre.bran@outlook.com

Alexa M Tullett	University of Alabama, Department of Psychology, Tuscaloosa, United States	alexa.tullett@gmail.com
Anna O. Kuzminska	University of Warsaw, Faculty of Management, Warsaw, Poland	akuzminska@wz.uw.edu.pl
Anthony J Krafnick	Dominican University, Department of Psychology, River Forest, United States	akrafnick@dom.edu
Anum Urooj	La Trobe University, Melbourne, Australia	ain.sonia@gmail.com
Ahmed Khaoudi	Mohammed V University in Rabat, Rabat, Morocco	ahmedkhaoudi@gmail.com
Afroja Ahmed	University of Limerick, Global MINDS, Department of Psychology, Limerick, Ireland	ahmedafroja25@gmail.com
Agata Groyecka-Bernard	University of Wroclaw, Institute of Psychology, Wroclaw, Poland<br>Johannes Gutenberg University, Social and Legal Psychology, Mainz, Germany	agata.groyecka@gmail.com
Adrian Dahl Askelund	Nic Waals Institute, Lovisenberg Diaconal Hospital, Oslo, Norway	adrian.askelund@gmail.com
Adeyemi Adetula	Université Grenoble Alpes, LIP/PC2S, Grenoble, France<br>Alex Ekwueme Federal University, Department of Psychology, Ndufu-Alike, Nigeria	adeyemiadetula1@gmail.com
Anabel Belaus	Universidad Nacional de Córdoba (UNC), Facultad de Psicología; Instituto de Investigaciones Psicológicas (IIPsi) - Consejo Nacional de Investigaciones Científicas y Técnicas (CONICET) - UNC, Córdoba, Argentina	abelaus@unc.edu.ar
Abdelilah Charyate	Ibn Tofail University, Higher College of Education & Training<br>Kenitra, Morocco	abdelilah.charyate@uit.ac.ma
Aaron L. Wichman	Western Kentucky University, Psychological Sciences Department, Bowling Green, United States	aaron.wichman@wku.edu
Alina Stoyanova	Sofia University St. Kliment Ohridski, Department of General, Experimental, Developmental, and Health Psychology, Sofia, Bulgaria	a.svilenova@gmail.com
Anna Greenburgh	University College London, Department of Experimental	a.greenburgh@ucl.ac.uk

	Psychology, London, United Kingdom	
Andrew G. Thomas	Swansea University, Psychology Department, Swansea, United Kingdom	research@agthomas.net
Alexios Arvanitis	University of Crete, Department of Psychology, Crete, Greece	a.arvanitis@uoc.gr
Patrick S Forscher	Université Grenoble Alpes, LIP/PC2S, Grenoble, France<br>Busara Center for Behavioral Economics, Nairobi, Kenya	schnarrd@gmail.com
Peter R Mallik	Ashland University, Department of Psychology, Ashland, United States	pmallik@ashland.edu
Nicholas A. Coles	Harvard University, Harvard Kennedy School, Boston, United States<br>Stanford University, Center for the Study of Language and Information, California, United States	ncoles797@gmail.com
Jeremy K. Miller	Willamette University, Department of Psychology, Salem, United States	millerj@willamette.edu
Hannah Moshontz	University of Wisconsin-Madison, Department of Psychology, Madison, United States	hmoshontz@gmail.com
Heather L. Urry	Tufts University, Department of Psychology, Medford, United States	heather.urry@tufts.edu
Hans IJzerman	Université Grenoble Alpes, Grenoble, France<br>Institut Universitaire de France, Paris, France	h.ijzerman@gmail.com
Dana M. Basnight-Brown	United States International University Africa, Nairobi, Kenya	dana.basnightbrown@usiu.ac.ke
Charles R. Ebersole	University of Virginia, Department of Psychology, Charlottesville, United States	cebersole@virginia.edu
Christopher R. Chartier	Ashland University, Department of Psychology, Ashland, United States	cchartie@ashland.edu
Erin M. Buchanan	Harrisburg University of Science and Technology, Harrisburg, United States	ebuchanan@harrisburgu.edu
Maximilian A. Primbs	Radboud University, Behavioural Science Institute, Nijmegen, Netherlands	maximilian.primbs@gmx.de

Note. Authors are presented in 3 tiers, with the first tier of five authors being the lead team, the last team being the lead admin team, and the middle tier being everyone who contributed to data collection,

translation, methodology, and administrative tasks. Within the first tier, authorship order corresponds to contribution. Within the second and third tiers, authorship order is arbitrary (i.e., by alphabetical order of email address) with the exception of the last author, which was determined by a vote among the admin team.

Contribution Statement

Conceptualization: T. Nguyen, N. Legate, N. Weinstein, A.C. Moller, L. Legault

Data Curation: P.S. Forscher, E.M. Buchanan

Formal Analysis: T. Nguyen, C.R. Ebersole

Funding Acquisition: P.S. Forscher, H. Ijzerman, C. Chartier, E.M. Buchanan

Investigation: Z. Vally, Z. Tajchman, A.N. Zsido, Z. Chen, I. Ziano, C.D. Ceary, Y. Lin, Y. Kunisato, Q. Xiao, X. Jiang, E. Yao, W.S. Ryan, J. Wilson, W. Jimenez-leal, W. Law, W. Collins, K.L. Richard, W. Unanue, M. Vranka, V. Lerche, V. Ankushev, V. Barkoukis, V. Schei, C. Depaola, U.S. Tran, S. Yeung, W. Hassan, T.J. Lima, T. Ostermann, T. Frizzo, T.E. Sverdrup, T. House, T. Gill, T. Jernsäther, T. Machin, M. Koptjevskaja-tamm, T.J. Hostler, T. Ishii, A. Studzinska, S.M. Janssen, S.E. Schulenberg, S. Tatachari, S. Azouaghe, P. Sorokowski, A. Sorokowska, X. Song, S. Morbée, D. Grigoryev, S. Daches, S.L. Levine, S.N. Geniole, S. Vračar, S. Massoni, S. Zorjan, E. Sarioguz, S.G. Alves, S. Pöntinen, S. Álvarez Solas, S. Ordoñez-Riaño, S. Batić Očovaj, S. Onie, S. Lins, S. Çoksan, A. Sacakli, S. Ruiz-Fernández, S. Fatahmodares, R.B. Walczak, R. Vilar, R. Doekemeijer, R.A. Cárcamo, R.M. Ross, R. Mccarthy, T. Ballantyne, E.C. Westgate, R. Gargurevich, R. Afhami, D. Ren, R.P. Monteiro, U. Reips, N. Reggev, R.J. Calin-jageman, R. London, R. Oliveira, M. Nedelcheva-Datsova, R. Rahal, T. Radtke, R. Searston, P. Zdybek, S. Chen, P. Wajanatinapart, J.T. Perillo, P. Kačmár, P. Macapagal, P. Szwed, P. Hanel, P.A. Forbes, P. Arriaga, N. Parashar, K. Papachristopoulos, P.S. Correa, C. Ogbonnaya, O. Bialobrzeska, N. Kiselnikova, N. Simonovic, N. Nock, C. Thogersen-Ntoumani, N. Ntoumanis, A. Hernandez, N. Albayrak-Aydemir, N. Say, A.B. Neubauer, N.I. Martin, N. Levy, N. Torunsky, N. Van Antwerpen, N. Van Doren, N. Sunami, N.R. Rachev, N.M. Majeed, N.S. Corral-Frías, N. Ouherrou, M.Y. Lucas, M. Pantazi, M.R. Vasilev, M. V. Ortiz, M.M. Butt, R. Muda, M.C. Tejada Rivera, M. Sirota, M. Seehuus, E. Smit, M. Parzuchowski, M. Toro, M. Hricova, M. Marszalek, M. Metz, M. Karekla, G. Mioni, L. Matos, M. Westerlund, M. Vdovic, M. Bialek, M. Anne, M. Misiak, M. Grinberg, M.F. Espinoza Barría, M.C. Mensink, M. Harutyunyan, M. Khosla, M. Adamkovič, M.F. Ribeiro, M. Terskova, M. Hruška, M. Martončik, M. Voracek, M. Frias-Armenta, M. Kowal, M. Roczniewska, P. Rentzelas, M. Braun Kohlová, M. Paruzel-Czachura, M. Romanova, M. Papadatou-Pastou, M. Magrin, M.V. Jones, M. Li, M.S. Ortiz, M. Manavalan, M. Kossowska, M.L.M. van Hooff, M.A. Varella, M. Standage, M. Nicolotti, M.F. Colloff, M. Bradford, L. Vaughn, L. A. Eudave, L. Vieira, L. Calderón Pérez, L.B. Lazarevic, L.M. Jaremka, E. Kushnir, G. Lins De Holanda Coelho, L. Ahlgren, F. Liga, L. Gunton, L. Volz, L. Boucher, L. Javela Delgado, L. Beatrix, K. Yu, J. Wachowicz, K. Desai, K. Barzykowski, M. Bayyat, L. Kozma, K. Evans, M.A. Koehn, K. Wolfe, K. Morris, K. Klevjer, K. Vezirian, K. Damnjanović, K. Schmidt, K. Moon, J. Kielińska, J.E. Cruz Vásquez, J. Chanal, J. Vargas-nieto, J.T. Roxas, J. Taber, J. Urriago-Rayo, J.M. Pavlacic, J. Benka, J. Bavolar, J.A. Soto, J.K. Olofsson, J.K. Vilsmeier, J. Czamanski-cohen, J.D. Moss, J. Boudesseul, J. Kamburidis, J.A. Joy-Gaba, J. Zickfeld, J.F. Miranda, E. Hristova, J. Milosevic Djordjevic, J.V. Valentova, J. Antfolk, J. Berkessel, J. Schrötter, J. Urban, J. Röer, J.O. Norton, J.R. Silva, J. Uttley, J.R. Kunst, I.L. Ndukaihe, A. Iyer, I. Vilares, A. Ivanov, I. Ropovik, I. Sarieva, I. Prusova, I. Pinto, I.A. Almeida, I. Dalgar, I. Zakharov, A. Arinze, K. Ihaya, I.D. Stephen, B. Gjoneska, H. Brohmer, H. Flowe, H. Godbersen, H. Kocalar, M.V. Hedgebeth, H. Manley, G. Kaminski, G. Nilsonne, G. Anjum, G.A. Travaglino, G. Feldman, G. Pfuhl, G. Marcu, G. Hofer, G. Banik, G. Bijlstra, F. Verbruggen, F.Y.H. Kung, F. Martela, F. Foroni, J. Forest, G. Singer, F. Mosannenzadeh, E. Marinova, E. Štrukelj, E. Baskin, E. Luis Garcia, E. Musser, E. Ahn, E. Pronizius, E.A. Jackson, E. Manunta, E. Agadullina, D. Šakan, O. Dujols, D. Dubrov, M. Willis, M. Tümer, I. Djamai, D. Herrera, D. Vega, H. Du, D. Mola, D. Chakarova, W.E. Davis, D. Holford, D. Lewis, D.C. Vaidis, D. Hausman Ozery, D. Zambrano Ricaurte, D. Storage, D. Sousa, D. Serrato Alvarez, D. Boller, A. Dalla Rosa, D. Marko, D. Moreau, C. Reeck, R.C. Correia, C.M. Whitt, C. Lamm, C. Singh Solorzano, C.C. von Bastian, C.A. Sutherland, C.L. Aberson, C.P. Niemiec, B. Soenens, M. Vansteenkiste, C. Reimer, C. Karashiali, C. Noone, F. Chiu, C. Eben, C. Brownlow, C. Karaarslan, N. Cellini, C. Esteban-serna, C. Reyna, C. Batres, C. Grano, J. Carpentier, C.H. Fu, B. Jaeger, C. Bundt, T. Bulut Allred, A. Bokkour, N. Bogatyreva, W.J. Chopik, B. Antazo, B. Behzadnia, M. Becker, W. Chou, H. Bai, B. Balci, P. Babinčák, B.J. Dixson, A. Mokady, H.B. Kappes, M. Atari, J. Aruta, A. Domurat, N. Arinze, A. Vatakis, A. Adiguzel, K. Ait El Arabi, A.A. Özdoğru, A. Olaya Torres, A. Theodoropoulou, A.P. Kassianos, A. Findor, A. Hartanto, A. Thibault Landry, A. Ferreira, A.C. Santos, A. De La Rosa-Gomez, A. Gourdon-Kanhukamwe, A. Karababa, A. Janak, A. Bran,

A.M. Tullett, A.O. Kuzminska, A.J. Krafnick, A. Urooj, A. Khaoudi, A. Ahmed, A. Groyecka-bernard, A. Askelund, A. Adetula, A. Belaus, A.C. Charyate, A.L. Wichman, A. Stoyanova, A. Greenburgh, A. Thomas, A. Arvanitis, P.S. Forscher, J.K. Miller, H. Urry, H. Ijzerman, E.M. Buchanan, M.A. Primbs

Methodology: C.R. Ebersole, A.C. Moller, M. Maniaci, R.M. Ryan, P. Arriaga, M.A. Silan, M.C. Mensink, J.S. Pickering, I. Ropovik, F. Azevedo, A. Thomas, H. Urry, D.M. Basnight-brown

Project Administration: C.R. Ebersole, Y. Jang, Y. Yamada, M. Vranka, V. Kovic, V. Križanić, T. Machin, S. Lewis, S. Meir Drexler, S. Morales Izquierdo, S. Tshonda, T. Biberauer, N. Reggev, R. Habte, P. Hanel, P. Arriaga, B. Paris, M.R. Vasilev, M. Alarcón Maldonado, M.A. Silan, M. Martončik, M. Oosterlinck, C.A. Levitan, L. Volz, L. Kozma, K. Barzykowski, K. Kirgizova, K. Thommesen, J.W. Suchow, J.E. Beshears, J. Antfolk, I. Sula, I.L. Pit, I. Dalgar, M. R. Dunn, A. Luxon, B. Gjoneska, H. Chuan-peng, M. Sharifian, H. Azab, G. Kaminski, G. Marcu, F. Azevedo, E. Štrukelj, E. Pronizius, J.L. Beaudry, D. Dunleavy, F. Chiu, N. Cellini, B. Ishkhanyan, L. Bylinina, T. Bulut Allred, M. Becker, A. Szabelska, A. Singh, A.P. Kassianos, A. Gourdon-Kanhukamwe, A. Todsen, A. Urooj, A. Ahmed, A. Askelund, A. Adetula, P.S. Forscher, P.R. Mallik, N.A. Coles, J.K. Miller, H. Moshontz, H. Urry, H. Ijzerman, D.M. Basnight-brown, C. Chartier, E.M. Buchanan, M.A. Primbs, E.L. Bradshaw

Resources: A.N. Zsido, M. Zrimsek, Z. Chen, Z. Gialitaki, Y. Jang, Y. Kunisato, Y. Yamada, X. Jiang, X. Du, E. Yao, W. Cyrus-lai, W. Law, M. Vranka, V. Schei, V. Križanić, V.H. Kadreva, V. Cubela Adoric, R. Houston, T.E. Sverdrup, M. Fedotov, T. Jernsäther, T. Rahman, M. Koptjevskaja-Tamm, T. Ishii, B. Szaszi, S. Adamus, L. Suter, S. Habib, A. Studzinska, D. Stojanovska, S. Stieger, S. Azouaghe, S. Lewis, S. Sinkolova, S. Meir Drexler, S. Akter, S. Massoni, E. Sarıoğuz, S. Morales Izquierdo, S.G. Alves, S. Pöntinen, S. Çoksan, A. Sacakli, S. Ruiz-Fernández, S.J. Geiger, R. Betlehem, R. Vilar, R.P. Monteiro, U. Reips, N. Reggev, R. Pourafshari, R. Oliveira, M. Nedelcheva-datsova, R.R. Ribeiro, R. Habte, S. Chen, P.G. Maturan, P. Kačmár, P. Hanel, P. Arriaga, B. Paris, O. Kácha, M. Bernardo, O. Campos, O. Niño Bravo, O.J. Galindo-Caballero, O. Bialobrzeska, N. Cohen, N. Johannes, N. Say, N. Sunami, N.R. Rachev, N. Schmidt, K. Nadif, N. Abbas, M. Pantazi, M.R. Vasilev, M. Kurfalı, M. Kabir, M. Parzuchowski, M. Alarcón Maldonado, M. Marszalek, G. Mioni, M.J. Bosma, M. Westerlund, M. Vdovic, M. Bialek, M.A. Silan, M. Capizzi, M. A. Kurfali, M. Harutyunyan, M. Korbmacher, M. Adamkovič, M. Hruška, M. Jansen, M. Čadek, M. Kowal, M. Topor, T. Paltrow, M. Roczniewska, M. Oosterlinck, M. Paruzel-czachura, M. Sabristov, M. Lund, M. Antoniadi, A. Muminov, M. Kossowska, M. Friedemann, M. Wielgus, L. Vieira, L. Sanabria Pineda, E. Kushnir, L. Anton-boicuk, G. Lins De Holanda Coelho, L. Ahlgren, C.A. Levitan, L. Micheli, L. Volz, M. Stojanovska, L. Samojlenko, L. Kaliska, L. Beatrix, L. Warmelink, L. Rojas-Berscia, J. Wachowicz, K. Barzykowski, L. Kozma, K. Kirgizova, B.B. Agesin, T. Korobova, K. Klevjer, K. Van Schie, K. Vezirian, K. Thommesen, K. Filip, K. Staniaszek, K. Grzech, K. Hoyer, K. Rana, K. Janjić, J. Kielińska, J. Beitner, J. Vargas-Nieto, J. Bavolar, J. Messerschmidt, J. Czamanski-cohen, J. Lee, J. Kamburidis, J. Zickfeld, J.P. Verharen, E. Hristova, J.E. Beshears, J. Milosevic Djordjevic, J. Bosch, J. Antfolk, J. Berkessel, J. Schrötter, J. Vintr, J.R. Kunst, I. Ropovik, I. Sula, I. Metin-orta, A. Bozdoc, I.L. Pit, I. Dalgar, I. Zakharov, K. Ihaya, B. Gjoneska, H. Kocalar, H. Chuan-peng, M. Sharifian, H. Akkas, H. Azab, G. Kaminski, G. Nilsonne, G. Czarnek, G. Marcu, G. Banik, G.A. Adetula, F. Muchembled, F. Azevedo, F. Mosannenzadeh, E. Marinova, E. Štrukelj, Z. Etebari, I. Van Steenkiste, E. Pronizius, E. Manunta, D. Šakan, P. Dursun, O. Dujols, D. Dubrov, M. Tümer, J.L. Beaudry, D. Popović, D. Dunleavy, I. Djamai, D. Krupić, D. Vega, D. Zambrano Ricaurte, D. Serrato Alvarez, A. Dalla Rosa, D. Dimova, D. Krupić, D. Marko, R.C. Correia, C. Singh Solorzano, C.C. von Bastian, C. Overkott, C. Wang, F. Chiu, C. Picciocchi, C. Karaarslan, N. Cellini, R. Li, C. Grano, C. Tamnes, B. Ishkhanyan, T. Bulut Allred, A. Bokkour, N. Bogatyreva, J. Shi, B. Antazo, M. Becker, B. Cocco, B. Hubena, B. Žuro, B. Aczel, E. Baklanova, A. Mokady, A. Szala, A. Szabelska, J. Aruta, A. Domurat, A. Modena, A. Adiguzel, A. Monajem, K. Ait El Arabi, A.A. Özdoğru, A. Skowronek, A. Penić Jurković, A.P. Kassianos, A. Findor, A. Thibault Landry, A.C. Santos, A. Gourdon-Kanhukamwe, A. Todsen, A. Karababa, A. Pilato, A. Bran, A. Khaoudi, A. Ahmed, A. Groyecka-Bernard, A. Askelund, A. Adetula, A. Stoyanova, E.M. Buchanan

Supervision: C.R. Ebersole, K. Thommesen, J.W. Suchow, M. Sharifian, A. Todsen, P.S. Forscher, P.R. Mallik, N.A. Coles, J.K. Miller, H. Moshontz, H. Urry, H. Ijzerman, D.M. Basnight-brown, C. Chartier, E.M. Buchanan, M.A. Primbs

Writing - Original Draft: T. Nguyen, N. Legate, N. Weinstein, A.C. Moller, L. Legault

Writing - Review and Editing: C.R. Ebersole, A.C. Moller, Z. Vally, Z. Tajchman, A.N. Zsido, M. Zrimsek, Z. Chen, I. Ziano, Z. Gialitaki, C.D. Ceary, Y. Jang, Y. Lin, Y. Kunisato, Y. Yamada, Q. Xiao, X. Jiang, X. Du, E. Yao, W.S. Ryan, J. Wilson, W. Cyrus-Lai, W. Jimenez-Leal, W. Law, W. Collins, K.L. Richard, W. Unanue, M. Vranka, V. Ankushev, V. Barkoukis, V. Schei, C. Depaola, V. Lerche, V. Kovic, V. Križanić, V.H. Kadreva, V. Cubela Adoric, U.S. Tran, S. Yeung, W. Hassan, R. Houston, T.J. Lima, T. Ostermann, T. Frizzo, T.E. Sverdrup, T. House, T. Gill, M. Fedotov, T. Jernsäther, T. Rahman, T. Machin, M. Koptjevskaja-Tamm, T.J. Hostler, T. Ishii, B. Szaszi, S. Adamus, L. Suter, S. Habib, A. Studzinska, D. Stojanovska, S.M. Janssen, S. Stieger, S.E. Schulenberg, S. Tatachari, S. Azouaghe, M. Vansteenkiste, P. Sorokowski, A. Sorokowska, X. Song, S. Morbée, S. Lewis, S. Sinkolova, D. Grigoryev, S. Meir Drexler, S. Daches, S.L. Levine, S.N. Geniole, S. Akter, S. Vračar, S. Massoni, S. Zorjan, E. Sarıoğuz, S. Morales Izquierdo, S. Tshonda, S.G. Alves, S. Pöntinen, S. Álvarez Solas, S. Ordoñez-Riaño, S. Batić Očovaj, S. Onie, S. Lins, T. Biberauer, S. Çoksan, A. Sacakli, S. Ruiz-Fernández, S.J. Geiger, S. Fatahmodares, R.B. Walczak, R. Betlehem, R. Vilar, R. Doekemeijer, R.A. Cárcamo, R.M. Ross, R. Mccarthy, T. Ballantyne, E.C. Westgate, R. Gargurevich, R.M. Ryan, P. Rentzelas, B. Soenens, R. Afhami, D. Ren, R.P. Monteiro, U. Reips, N. Reggev, E. Smit, L. Matos, R.J. Calin-jageman, R. Pourafshari, R. London, R. Oliveira, M. Nedelcheva-Datsova, R. Rahal, R.R. Ribeiro, T. Radtke, R. Searston, R. Habte, P. Zdybek, S. Chen, P. Wajanatinapart, P.G. Maturan, J.T. Perillo, P.M. Isager, P. Kačmár, P. Macapagal, M. Maniaci, P. Szwed, P. Hanel, P.A. Forbes, P. Arriaga, B. Paris, T. Paltrow, N. Parashar, K. Papachristopoulos, P.S. Correa, O. Kácha, M. Bernardo, O. Campos, O. Niño Bravo, O.J. Galindo-caballero, C. Ogbonnaya, O. Bialobrzeska, N. Kiselnikova, N. Simonovic, N. Cohen, N. Nock, A. Hernandez, C. Thogersen-Ntoumani, N. Ntoumanis, N. Johannes, N. Albayrak-Aydemir, N. Say, A.B. Neubauer, N.I. Martin, N. Levy, N. Torunsky, N. Van Antwerpen, N. Van Doren, N. Sunami, N.R. Rachev, N.M. Majeed, N. Schmidt, K. Nadif, N.S. Corral-Frías, N. Ouherrou, N. Abbas, M. Pantazi, M.Y. Lucas, M.R. Vasilev, M.V. Ortiz, M.M. Butt, M. Bayyat, M. Kurfalı, M. Kabir, R. Muda, M.C. Tejada Rivera, M. Sirota, M. Seehuus, M. Parzuchowski, M. Toro, M. Hricova, M. Alarcón Maldonado, M. Metz, M. Marszalek, M. Karekla, G. Mioni, M.J. Bosma, M. Westerlund, M. Vdovic, M. Bialek, M.A. Silan, M. Anne, M. Misiak, M. Grinberg, M. Capizzi, M.F. Espinoza Barría, M. A. Kurfali, M.C. Mensink, M. Harutyunyan, M. Khosla, M. Korbmacher, M. Adamkovič, M.F. Ribeiro, M. Terskova, M. Hruška, M. Martončik, M. Jansen, M. Voracek, M. Čadek, M. Frias-Armenta, M. Kowal, M. Topor, M. Roczniewska, M. Oosterlinck, M. Braun Kohlová, M. Paruzel-Czachura, M. Sabristov, M. Romanova, M. Papadatou-Pastou, M. Lund, M. Antoniadi, M. Magrin, M.V. Jones, M. Li, M.S. Ortiz, M. Manavalan, A. Muminov, M. Kossowska, M. Friedemann, M. Wielgus, M.L.M. van Hooff, M.A. Varella, M. Standage, M. Nicolotti, M.F. Colloff, M. Bradford, L. Vaughn, L. A.Eudave, L. Vieira, L. Sanabria Pineda, L. Calderón Pérez, L.B. Lazarevic, L.M. Jaremka, E. Kushnir, L. Anton-boicuk, G. Lins De Holanda Coelho, L. Ahlgren, F. Liga, C.A. Levitan, L. Micheli, L., L. Gunton, Volz, M. Stojanovska, L. Boucher, L. Samojlenko, L. Javela Delgado, L. Kaliska, L. Beatrix, L. Warmelink, L. Rojas-berscia, K. Yu, J. Wachowicz, K. Desai, K. Barzykowski, L. Kozma, K. Evans, K. Kirgizova, B.B. Agesin, M.A. Koehn, K. Wolfe, T. Korobova, K. Morris, K. Klevjer, K. Van Schie, K. Vezirian, K. Damnjanović, K. Thommesen, K. Schmidt, K. Filip, K. Staniaszek, K. Grzech, K. Hoyer, K. Moon, K. Rana, K. Janjić, J.W. Suchow, J. Kielińska, J.E. Cruz Vásquez, J. Chanal, J. Beitner, J. Vargas-Nieto, J.T. Roxas, J. Taber, J. Urriago-Rayo, J.M. Pavlacic, J. Benka, J. Bavolar, J.A. Soto, J.K. Olofsson, J.K. Vilsmeier, J. Messerschmidt, J. Czamanski-cohen, J.D. Moss, J. Boudesseul, J. Lee, J. Kamburidis, J.A. Joy-Gaba, J. Zickfeld, J.F. Miranda, J.P. Verharen, E. Hristova, J.E. Beshears, J. Milosevic Djordjevic, J. Bosch, J.V. Valentova, J. Antfolk, J. Berkessel, J. Schrötter, J. Urban, J. Röer, J.O. Norton, J.R. Silva, J.S. Pickering, J. Vintr, J. Uttley, J.R. Kunst, I.L. Ndukaihe, A. Iyer, I. Vilares, A. Ivanov, I. Ropovik, I. Sula, I. Sarieva, I. Metin-orta, I. Prusova, I. Pinto, A. Bozdoc, I.A. Almeida, I.L. Pit, I. Dalgar, I. Zakharov, A. Arinze, K. Ihaya, I.D. Stephen, B. Gjoneska, H. Brohmer, H. Flowe, H. Godbersen, H. Kocalar, M.V. Hedgebeth, H. Chuan-peng, M. Sharifian, H. Manley, H. Akkas, H. Azab, G. Kaminski, G. Nilsonne, G. Anjum, G.A. Travaglino, G. Feldman, G. Pfuhl, G. Czarnek, G. Marcu, G. Hofer, G. Banik, G.A. Adetula, G. Bijlstra, F. Verbruggen, F.Y.H. Kung, F. Martela, F. Foroni, J. Forest, G. Singer, F. Muchembled, F. Azevedo, F. Mosannenzadeh, E. Marinova, E. Štrukelj, Z. Etebari, E. Baskin, E. Luis Garcia, E. Musser, I. Van Steenkiste, E. Ahn, E. Pronizius, E.A. Jackson, E. Manunta, E. Agadullina, D. Šakan, P. Dursun, O. Dujols, D. Dubrov, M. R. Dunn, M. Willis, M. Tümer, J.L. Beaudry, D. Popović, D. Dunleavy, I. Djamai, D. Krupić, D. Herrera, D. Vega, H. Du, D. Mola, D. Chakarova, W.E. Davis, D. Holford, D. Lewis, D.C. Vaidis, D. Hausman Ozery, D. Zambrano Ricaurte, D. Storage, D. Sousa, D. Serrato Alvarez, D. Boller, A. Dalla Rosa, D. Dimova, D. Krupić, D. Marko, D. Moreau, C. Reeck, R.C. Correia, C.M. Whitt, C. Lamm, A. Luxon C. Singh Solorzano, C.C. von Bastian, C.A. Sutherland, C. Overkott, C.L. Aberson, C. Wang, C.P. Niemiec, C.

Reimer, C. Karashiali, C. Noone, F. Chiu, C. Picciocchi, C. Eben, C. Brownlow, C. Karaarslan, N. Cellini, C. Esteban-serna, C. Reyna, C. Batres, R. Li, C. Grano, J. Carpentier, C. Tamnes, C.H. Fu, B. Ishkhanyan, L. Bylinina, B. Jaeger, C. Bundt, T. Bulut Allred, A. Bokkour, N. Bogatyreva, J. Shi, W.J. Chopik, B. Antazo, B. Behzadnia, M. Becker, B. Cocco, W. Chou, B. Hubena, B. Žuro, B. Aczel, E. Baklanova, H. Bai, B. Balci, P. Babinčák, B.J. Dixson, A. Mokady, H.B. Kappes, M. Atari, A. Szala, A. Szabelska, J. Aruta, A. Domurat, N. Arinze, A. Modena, A. Vatakis, A. Adiguzel, A. Monajem, K. Ait El Arabi, A.A. Özdoğru, A. Olaya Torres, A. Theodoropoulou, A. Skowronek, A. Penić Jurković, A. Singh, A.P. Kassianos, A. Findor, A. Hartanto, A. Thibault Landry, A. Ferreira, A.C. Santos, A. De La Rosa-gomez, A. Gourdon-Kanhukamwe, A. Todsen, A. Karababa, A. Janak, A. Pilato, A. Bran, A.M. Tullett, A.O. Kuzminska, A.J. Krafnick, A. Urooj, A. Khaoudi, A. Ahmed, A. Groyecka-Bernard, A. Askelund, A. Adetula, A. Belaus, A.C. Charyate, A.L. Wichman, A. Stoyanova, A. Greenburgh, A. Thomas, A. Arvanitis, P.S. Forscher, P.R. Mallik, N.A. Coles, J.K. Miller, H. Moshontz, H. Urry, H. Ijzerman, D.M. Basnight-brown, C. Chartier, E.M. Buchanan, M.A. Primbs, E.L. Bradshaw

Contributions listed here reflect documented contributions to the project. People who helped with translation are listed as Resources contributors; people who helped with data collection are listed as Investigation contributors.

There were additional contributors to this project who declined to be included as authors or did not reply to requests to provide information and approval. These people served in various roles, including Resources, Project Administration, Methodology, and Investigation.
